# Carrier screening by next‐generation sequencing: health benefits and cost effectiveness

**DOI:** 10.1002/mgg3.204

**Published:** 2016-01-29

**Authors:** Mohammad Azimi, Kyle Schmaus, Valerie Greger, Dana Neitzel, Robert Rochelle, Tuan Dinh

**Affiliations:** ^1^ Evidera450 Sansome StreetSuite 650San FranciscoCA; ^2^ Good Start Genetics, Inc.237 Putnam Ave.CambridgeMA

**Keywords:** Carrier screening, cost effectiveness, genotyping, next‐generation sequencing

## Abstract

**Background:**

Compared with conventional genotyping, which typically tests for a limited number of mutations, next‐generation DNA sequencing (NGS) provides increased accuracy for carrier screening. The objective of this study was to evaluate the cost effectiveness of carrier screening using NGS versus genotyping for 14 of the recessive disorders for which medical society guidelines recommend screening.

**Methods:**

Data from published literature, population surveys, and expert opinion were used to develop a decision tree model capturing decisions and outcomes related to carrier screening and reproductive health.

**Results:**

Modeling a population of 1,000,000 couples that was representative of the United States population and that contained 83,421 carriers of pathogenic mutations, carrier screening using NGS averted 21 additional affected births as compared with genotyping, and reduced costs by approximately $13 million. As compared with no screening, NGS carrier screening averted 223 additional affected births. The results are sensitive to assumptions regarding mutation detection rates and carrier frequencies in multiethnic populations.

**Conclusion:**

This study demonstrated that NGS‐based carrier screening offers the greater benefit in clinical outcomes and lower total healthcare cost as compared with genotyping.

## Introduction

It is estimated that Mendelian disorders collectively account for 10% of infant mortality and 20% of pediatric hospitalizations (Kumar et al. 2001). Carrier screening, accompanied by genetic counseling, has been demonstrated to significantly reduce incidences of recessive genetic disorders, for example, cystic fibrosis, Gaucher disease, and Tay–Sachs disease in individuals of Ashkenazi Jewish descent (Kronn et al. 1998; Grody et al. 2013). Carrier screening provides individuals with information about their reproductive risks prior to or during pregnancy by identifying gene mutations associated with autosomal recessive or X‐linked disorders. The American Congress of Obstetricians and Gynecologists (2009, 2011) (ACOG: in Committee Opinions No. 442 [2009] and 486 [2011]) and the American College of Medical Genetics and Genomics (ACMG: in a 2006 technical guideline and a 2013 position statement), among others, recommend screening for cystic fibrosis for all women of reproductive age, and screening for additional disorders, if indicated by family history, the couple's carrier status, or ethnicity (Amos et al. 2006, American Congress of Obstetricians and Gynecologists, 2009, 2011, Grody et al. 2013). (It is not within the scope of this paper to comment on the appropriateness of these or other related guidelines.)

Due to cost considerations and various technical barriers, traditional carrier screening assays are designed to identify only the most common mutations within a gene, rather than all known disease‐causing mutations. While this approach is effective in detecting specific mutations in specific populations (e.g., sickle cell disease in African Americans), it proves to be suboptimal for populations of mixed or unknown ethnicities. The advent of massively parallel next‐generation DNA sequencing (NGS) technologies has provided opportunities to radically improve strategies for carrier screening. Compared with conventional genotyping‐based carrier screening, which, due to numerous cost and technical limitations, is typically designed to detect a limited number of mutations for each disease, NGS provides increased accuracy (Grody et al. 2013). Therefore, NGS could facilitate a transition from targeted analysis of specific genes to a strategy of simultaneously testing a significantly larger number of alleles. In summary, the efficiency of NGS allows the inclusion of many more mutations per disease than is feasible with traditional genotyping‐based panels, resulting in higher carrier detection rates.

The excellent analytical accuracy (both sensitivity and specificity) and operational feasibility of NGS for carrier screening have been demonstrated in several recent studies (Bell et al. 2011; Hallam et al. 2014; Umbarger et al. 2014). Specifically, Hallam et al. (2014) used high‐throughput NGS to screen 11,691 patients visiting assisted reproductive technology (ART) centers and identified 449 mutant alleles (447 in carriers and 2 in an affected individual), and, in total, 87 distinct pathogenic mutations in 14 different genes. Most importantly, about one quarter of the mutations found were not included in traditional genotyping panels, including 16 known pathogenic mutations unique to the NGS panel, and novel truncating mutations in several genes. Similarly, Davie et al. (2015) evaluated 48,761 clinical samples and demonstrated that NGS‐based tests routinely detected common pathogenic variants among the 14 disorders, as well as numerous less common pathogenic variants that would not be detected by traditional screening assays routinely used by in vitro fertilization (IVF) centers. More specifically, 2309 (4.7%) patients were found to be carriers of 320 distinct pathogenic variants among the 14 disorders and 226 (63.1%) of those distinct pathogenic variants were either uncommon or never‐before reported, that is, unique to NGS. Of the 2309 carriers detected, 15.9–22.3% would have been missed by other major laboratories using traditional genotyping. In addition, whether employing genotyping or NGS for carrier screening, the laboratory must also assure that the variants the tests are designed to detect are indeed pathogenic, or else risk an unacceptably low specificity (i.e., false positives), which can lead to higher overall costs. Perreault‐Micale et al. (2015) detailed a rigorous process of evaluating, cataloging, and curating only pathogenic mutations, thereby taking care not to include variants of unknown clinical significance, or VUSs, in its assays.

The objective of the present study was to evaluate the cost effectiveness of using NGS for carrier screening instead of traditional genotyping. Since there is no empirical data available at this time, we developed a robust mathematical model to estimate the health and economic outcomes of using NGS versus genotyping technologies for carrier screening of 14 recessive disorders recommended for screening by ACOG, ACMG, and/or various Ashkenazi Jewish advocacy groups (Richards et al. 2002, Watson et al. 2004; Gross et al. 2008; American Congress of Obstetricians and Gynecologists, 2009, 2011; Scott et al. 2010; Victor Center, 2015). The model accounts for all decisions and outcomes relevant to carrier screening and reproductive health. Our goal was to determine (1) whether carrier screening by NGS improves health and economic outcomes as compared with that by traditional genotyping (which often looks for small sets of mutations for each disease) and (2) whether carrier screening by NGS is cost‐effective as compared to no screening at all.

## Materials and Methods

### Ethical compliance

Given that our study involved only the creation and use of a decision tree model that was based on data from published literature, population surveys, and expert opinion, and that our study involved no human or animal subjects, IRB approval was not required.

### The model

**Figure 1:**
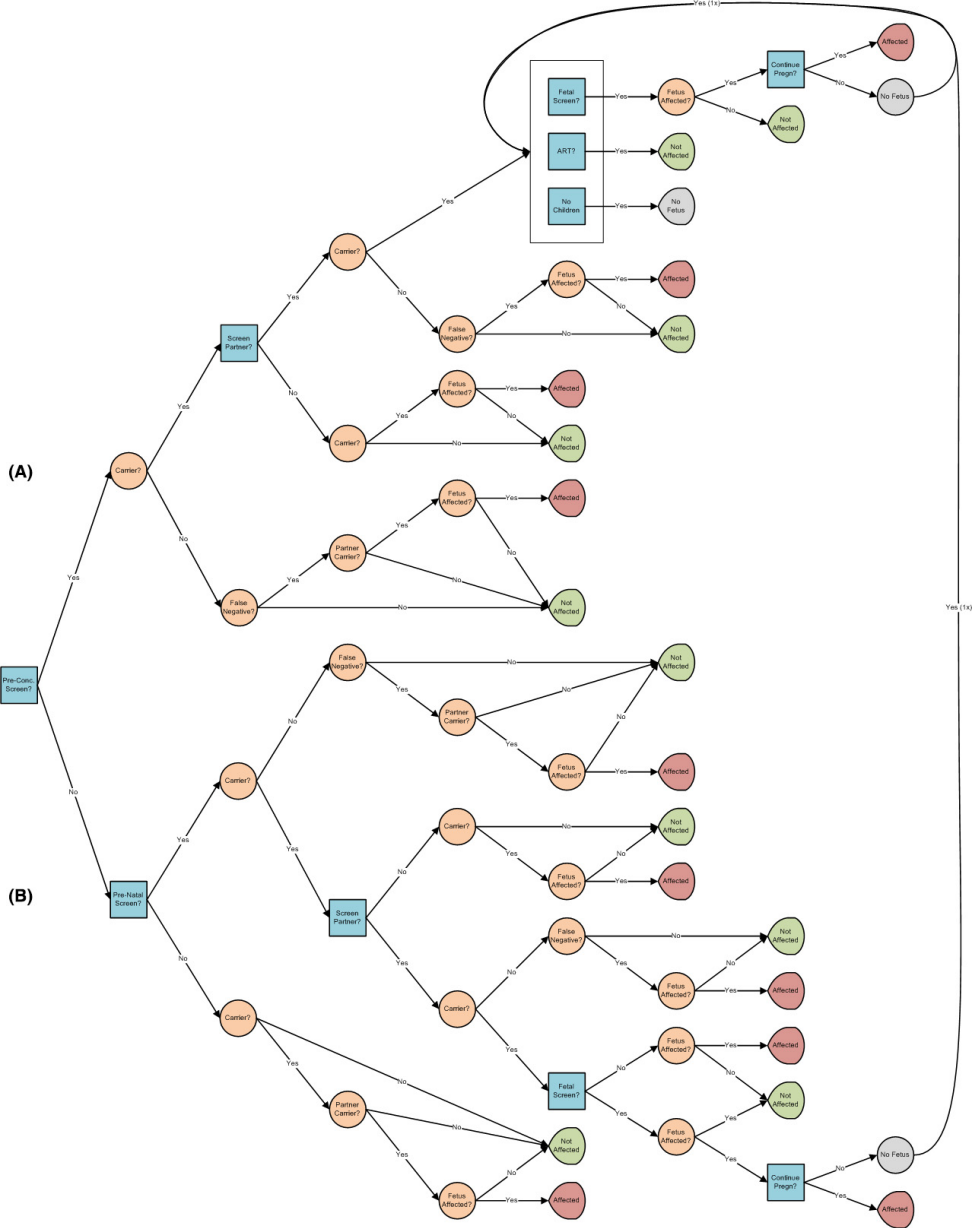
Decision tree: (A) preconception branch and (B) prenatal and no screening branches.

The model used a decision tree to evaluate health and economic outcomes following decisions related to carrier screening (see Fig. 1A and B). The decision tree was constructed based on interviews with experts with deep knowledge in carrier screening and reproductive health. The decision tree includes major decision nodes, clinical outcomes, and costs relevant to reproductive health and carrier screening, and captures both intended and unintended consequences associated with disorder occurrence and patient decisions regarding preventing or remedying such occurrences. Decision tree analyses captured carrier prevalence by disorder, patient ethnicity, screening detection rates, healthcare processes, patient behaviors, costs, and health utilities, among others.

Three categories of outcomes of reproductive health are predicted by the model:

- Birth of an unaffected child, born without a disorder (the child can be either genetically wild type or a carrier).
- Birth of an affected child, born with a disorder.
- Other, that is, instances where couples decide to not conceive, or decide to pursue adoption, use donor egg or sperm, preimplantation genetic diagnosis (PGD) on embryos, and/or use other interventions postconception or traditional prenatal screening via chorionic villus sampling or amniocentesis.

The decision tree consists of three major branches related to three types of pregnancies as follows (Fig. 1A and B):

1. *Pregnancies which utilize preconception carrier screening* (Fig. 1A): In this branch, one or both partners agree to carrier screening. (The model accounts for the possibility that a partner is either not available for or does not agree to carrier screening.) Following a negative screen of either partner, there is a residual risk that one or more mutations went undetected and the fetus will be a carrier or affected with the disorder. If both partners are identified as carriers, the partners have the following options: (1) conceiving at risk, with or without performing follow‐up fetal screening for the genetic disorder, with the option of terminating the pregnancy if the fetus is affected, (2) employing ART with or without PGD analysis of candidate embryos, or (3) deciding not to conceive (which includes the option to adopt) (Snowdon and Green 1997).
2. *Pregnancies which utilize prenatal carrier screening* (Fig. 1B): A significant proportion of pregnancies are either not planned or occur naturally prior to prenatal carrier screening. Couples with this type of pregnancy who are identified as carriers are offered postconception carrier screening. As a result, some pregnancies may be terminated and followed by loss replacement. (That is, based on their screening results, couples may opt to terminate a pregnancy, then decide to try to conceive again, in hopes that this next pregnancy would result in a healthy, nonaffected infant. Our model captures the costs associated not only with terminating a pregnancy but also with attempting, succeeding, or failing with another pregnancy following the termination of that first pregnancy.)
3. *Pregnancies which do not utilize any genetic screening* (Fig. 1B): In this branch of the decision tree, the model calculates the number of affected and unaffected children born to parents who decline any form of genetic screening.

Table S1 in Appendix S1 enumerates the inputs into and outcomes associated with each branch of the decision tree.

### Population generation

Using data from the literature and publicly available datasets (Table 1 and Appendix S1), we created a virtual population of 1,000,000 couples, representative of the United States population. The distribution of races and ethnicities are based on U.S. Census data. The mortality rates for unaffected populations are based on data provided by the Centers for Disease Control and Prevention (Arias 2014).

**Table 1:** Key parameters, assumptions, modeling approaches, and sources

Model parameter	Assumptions and approaches	Sources
*Decisions related to carrier screening*		
Decisions of couples identified as carriers during preconception screening	Probability of conceiving at risk and screening the fetus postconception: 50% (46–54%) Probability of pursuing assisted reproductive technology (egg/sperm donor or IVF with PGD): 30% (25–35%) Not pursue conception: 20% (11–29%)	Snowdon and Green (1997)
Decision of couples identified as carriers during prenatal carrier screening	To screen fetus: 80% (50–100%)	Rowley et al. (1998)
Decision following identification of affected fetus	To terminate pregnancy: 75% (50–100%)	Rowley et al. (1998), Brock (1996), Scotet et al. (2000, 2003)
Decision following identification of a carrier in one partner	To screen partner: 85% (50–100%)	Rowley et al. (1998), Radhakrishnan et al. (2008)
*Population characteristics*		
Ethnic distribution	Non‐Hispanic White: 65% Hispanic: 16% African American: 12% Asian: 5% Ashkenazi Jewish: 2%	American Congress of Obstetricians and Gynecologists (2009)
Mutation carrier rate	See Appendix S1	American Congress of Obstetricians and Gynecologists (2011), Scott et al. (2010), Amos et al. (2006), Chou et al. (2002), Kaback and Desnick (1999)
*Life expectancy*		
Life expectancy of healthy offspring	Based on United States Life Tables from the Centers for Disease Control and Prevention	Arias (2014)
Life expectancy of affected offspring	See Appendix S1	
*Costs*		
Carrier screening (both NGS and genotyping)	$500 ($300–$800)	Averaged from an author survey of U.S. commercial genetic test providers
Pregnancy termination	$2614 ($1960–$3267)	Rowley et al. (1998)
Assisted reproductive technology	$41,132 ($30,489–$51,415)	Brock (1996): based on the utilization weighted cost of the typical number of IVF cycles required for a live birth (fresh transfers, frozen‐thawed transfers, and associated medication costs), ICSI, assisted hatching, blastocyst culture, and cryopreservation.
Fetal screening	$1500 ($1000–$2000)	Song et al. (2013), Benn et al. (2013), Garfield and Armstrong (2012)
*Test performance*		
Genotyping detection rates and NGS mutation detection rates	See Appendix S1	Scott et al. (2010), Counsyl (2010), Ben‐Yosef et al. (2003), Edelmann et al. (2002), Ekstein et al. (2004), Heim et al. (2001), Ki et al. (2004), Ness et al. (2003), Nestorowicz et al. (1996), Park et al. (2010)

ICSI, intracytoplasmic sperm injection; IVF, in vitro fertilization; NGS, next‐generation DNA sequencing; PGD, preimplantation genetic diagnosis.

### Disease models

We developed models for 14 genetic diseases: Bloom's syndrome, Canavan disease, cystic fibrosis, dihydrolipoamide dehydrogenase deficiency, familial dysautonomia, familial hyperinsulinism, Fanconi anemia group C, glycogen storage disease type 1a, maple syrup urine disease type 1A/1B, mucolipidosis type IV, Niemann–Pick disease type A/B, Tay–Sachs disease, Usher syndrome type IF, and Usher syndrome type III. These are among the most prevalent Mendelian disorders and have been recommended for carrier screening by one or more professional associations, including ACOG, ACMG, and various Ashkenazi Jewish advocacy groups (American Congress of Obstetricians and Gynecologists, 2009, 2011, Grody et al. 2013). Estimates of mutation carrier rates and life expectancy of individuals affected by the disorders are based on synthesis of published literature and summarized in Appendix S1.

### Mutation detection rates

Mutation detection rates for genotyping‐based assays (or other assays that can detect only a limited number of mutations) are estimated from a survey of the U.S. commercial providers of carrier screening. Because NGS technology is relatively new, data on mutation detection rates are currently limited. We developed a model to estimate the detection rate of NGS for each recessive disorder by taking into account the fact that NGS detects not only so‐called common mutations that are included in smaller genotyping panels, but also less frequent mutations that are often excluded from typical genotyping panels (Appendix S1, Section C). NGS allows for the detection of many more mutations than traditional genotyping‐based carrier screens, while still detecting so‐called common mutations (Hallam et al. 2014; Perreault‐Micale et al. 2014). As a result, NGS is expected to yield higher detection rates than older, traditional genotyping approaches. More specifically, we used data from a database of 71,070 patients who underwent NGS‐based carrier screening in the clinical setting. Table S7 in Appendix S1 compares the number of mutation carriers detected by NGS in this population against those who would be detected by different traditional genotyping assays. Of the 3093 carriers detected, 11.0–25.8% would have been missed by other major laboratories using traditional genotyping. This is consistent with a recent multiethnic study in which a quarter of the mutations detected by NGS are not included in traditional, limited, mutation panels (Hallam et al. 2014). Our model predicts that, for a large population, NGS will identify 10% or more mutation carriers than traditional genotyping, which is also consistent with the improvements calculated using data from the clinical database of 71,070 patients described in Table S7.

Mutation detection rates for both genotyping‐based assays and NGS are summarized in Section C of Appendix S1 for other diseases. With the exception of cystic fibrosis, there is limited data on mutation detection rates available for ethnicities other than the Ashkenazi Jewish, so we estimated two mutation detection rates, one for individuals of Ashkenazi Jewish descent and one for other ethnicities as a group.

### Cost effectiveness

The model takes into account the direct medical costs associated with carrier screening and treatments of recessive disorders. These costs were primarily based on literature (Table 1 and Appendix S1). All costs were adjusted to 2014 values. A U.S. health plan perspective was used, with costs, benefits, and life years (LY) discounted 3%, and with adherence to other recommendations of the International Society for Pharmacoeconomics and Outcomes Research (Briggs et al. 2012; Caro et al. 2014).

### Study design

We conducted a simulation in which 1,000,000 simulated couples, representative of the general U.S. population, were exposed to three carrier screening strategies: (1) no screening, (2) carrier screening by traditional genotyping, and (3) carrier screening by NGS. In the two groups that received carrier screening, couples testing positive for one or more mutations were offered appropriate follow‐up options, depending on their reproductive status. The model accounts for the facts that not all partners are available for or agree to carrier screening, and that, after a negative screen of either partner, there is a residual risk that a mutation went undetected by screening and, subsequently, that the fetus will be a carrier of or affected with the disorder.

### Parameters and assumptions

Parameters, base case values, and assumptions used to inform the model were drawn from the literature and publicly available datasets to the greatest extent possible (Table 1 and Appendix S1). Where data were lacking, these values were estimated using conservative assumptions, by consensus among the study authors and external authorities with expertise on the disorders of interest, and appropriate sensitivity analyses were performed.

For this model, we assumed that the costs of carrier screening by NGS and traditional genotyping are equal. Because health plans currently bear most or all of the financial burden of carrier screening, it is most relevant to examine the costs of carrier screening to the health plan. For a product that is not covered by a health plan to become covered by that plan – and, therefore, widely available to physicians and their patients – it will need to match an incumbent product's price. (If one product is priced significantly higher than another, this could affect cost‐effectiveness metrics dramatically. However, that higher cost would also lead to a health plan policy that excludes that product from its network.) Due to these competitive forces and the purchasing power of the health plans, for the purposes of this analysis, then, it is assumed that all products’ prices to health plans eventually converge on the same price point.

### Sensitivity analysis

We performed a one‐way sensitivity analysis to quantify the influence of model parameters on cost‐effectiveness results. We varied each model parameter within a range representing plausible upper and lower limits. The ranges of the model parameters are based on literature and summarized in Table 1 and Appendix S1. To explore the variations in model predictions due to uncertainties in parameter estimations and the interactions between parameters, we conducted a probabilistic sensitivity analysis by sampling the model parameters concurrently from their probable distributions.

## Results

**Figure 2:**
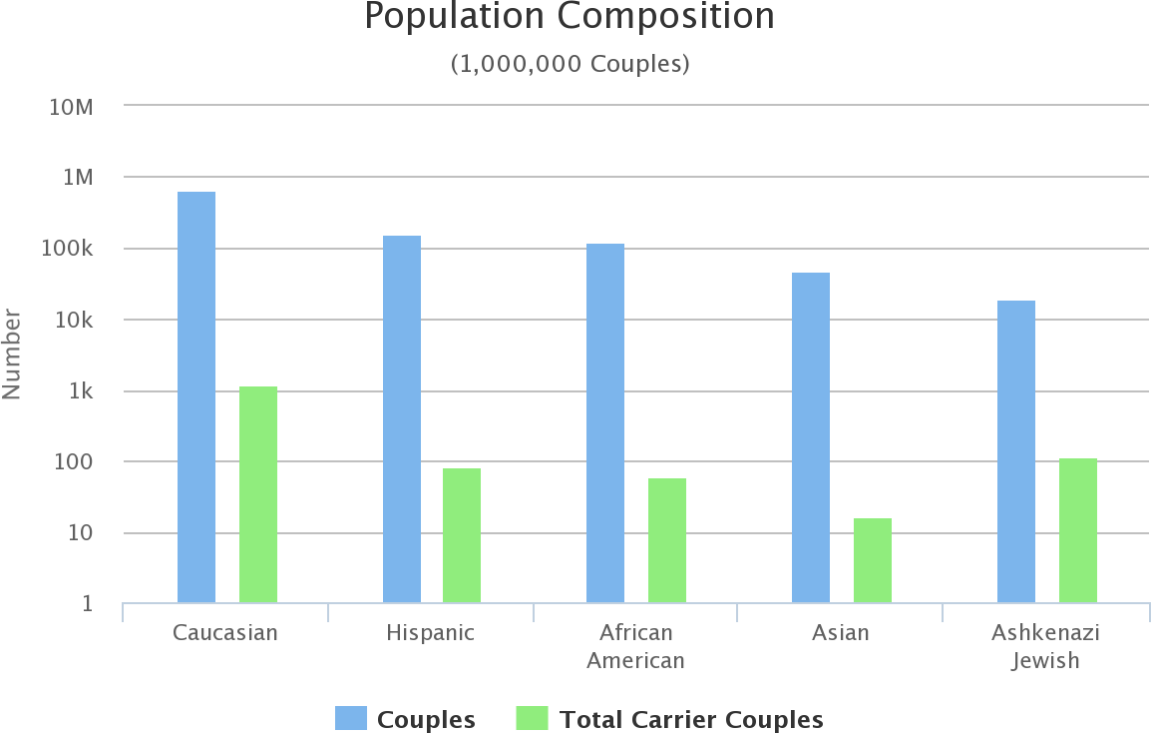
Projected composition of carrier status by ethnicity (simulated data), all disorders considered. Note the logarithmic *y*‐axis which shows orders of magnitude difference between number of couples and carrier couples.

Table 2 summarizes the health outcomes and costs for 1,000,000 simulated couples representative of the U.S. population that were exposed to the three carrier screening strategies. The model predicted that there would be 1457 couples, or one couple in 686, in which both partners would be carriers of mutations that cause the same disorder. The distribution of carriers in different ethnicities is illustrated in Figure 2. As compared with no screening, carrier screening by NGS reduced incidence of affected cases by 61% (Table 2). Lifetime treatment costs of the 14 recessive disorders were reduced by 66%. The majority of savings on treatment costs came from cystic fibrosis. Specifically, lifetime treatment costs of cystic fibrosis were reduced from $382.6 million to $123.2 million. The cost savings of treatments of recessive disorders were offset by the costs of ART, pregnancy termination, and fetal testing.

**Table 2:** Summary of results

Outcomes	No screening	Carrier screening by genotyping	Carrier screening by NGS
Population			
Number of couples		1,000,000	
Number of couples with at least one carrier		83,421	
Couples with a single carrier		81,964	
Couples with two carriers of the same disorder		1457	
Affected births	364	162	141
Number of affected births averted	0	202	223
LYs gained as compared with no screening	0	7918	8636
Costs			
Lifetime treatment costs of 14 recessive disorders	$415 million	$159 million	$140 million
ART cost + Termination cost + Traditional prenatal screening of the fetus	$0 million	$5.3 million	$4.7 million
Genetic screening costs	$0	$519 million	$525 million
Total lifetime healthcare costs related to recessive disorders (including ART, pregnancy termination, fetal screening)	$415 million	$683 million	$670 million
Cost effectiveness			
Cost per LY gained as compared with no screening		$33,812	$29,498
Cost per affected birth avoided as compared to no screening		$1.33 million	$1.14 million

ART, assisted reproductive technology; LY, life year; NGS, next‐generation DNA sequencing.

**Figure 3:**
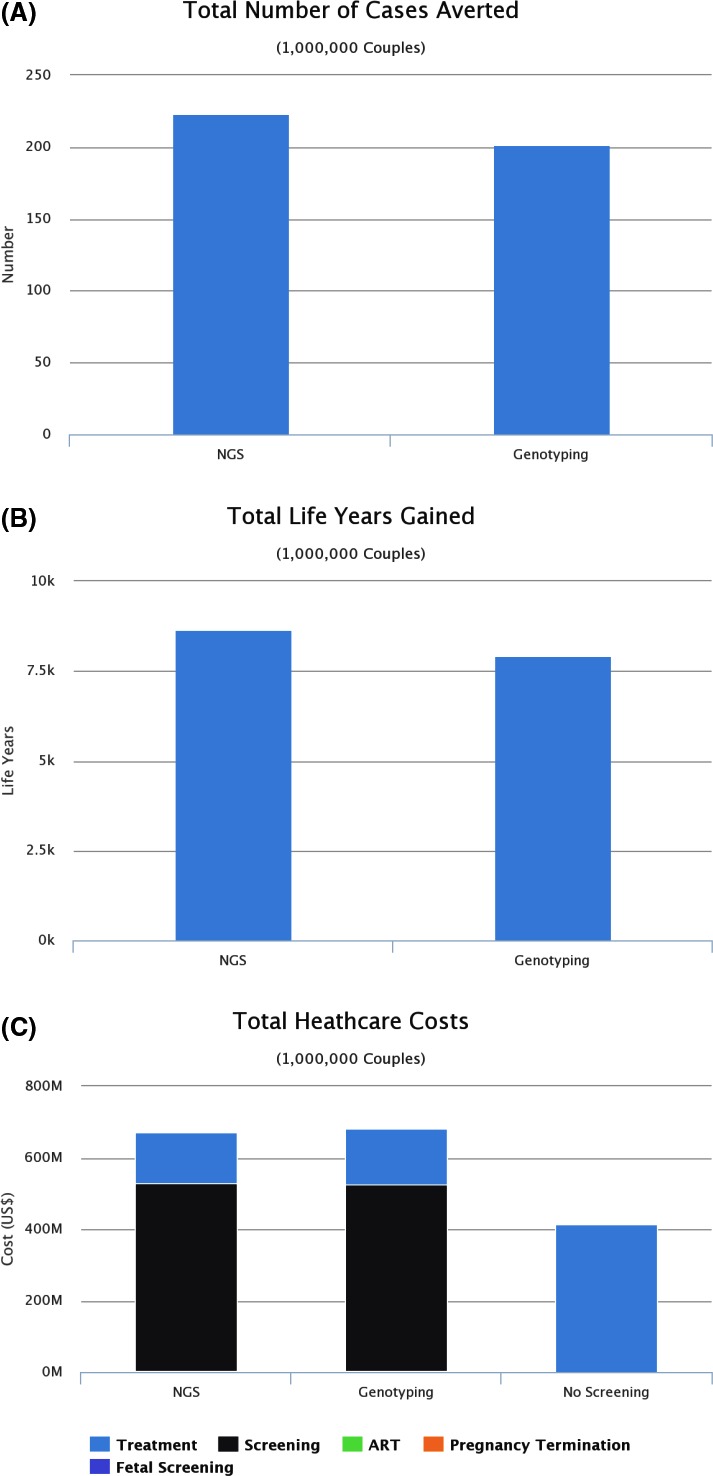
(A) Number of affected births prevented using next‐generation DNA sequencing (NGS) versus genotyping for carrier screening (simulated data). (B) Total life years gained from cases of genetic disorders averted (simulated data). (C) Healthcare costs for different screening scenarios (simulated data).

As compared with targeted disease screening using traditional genotyping, the NGS‐based strategy resulted in prevention of 21 (or 13%) additional affected births (Fig. 3A) and a gain of approximately 718 LYs (Fig. 3B). Although the total initial cost of genetic screening for the NGS strategy is larger ($525 million vs. $519 million) – for reasons such as increased mutation detection rates in the first partner screened leading to subsequent partner screening – its overall lifetime cost is approximately $13 million less than the genotyping strategy ($670 million vs. $683 million; Fig. 3C), as the savings in treatments of genetic disorders are greater than the additional costs associated with genetic screening. It is clear that NGS is the dominant strategy, offering the most cost‐effective option as compared with both no screening and traditional genotyping.

### Sensitivity analysis

Figures S1 and S2 and Table S11 in Appendix S1 summarizes the results of a single‐parameter sensitivity analysis. The sensitivity analysis indicates that parameters related to cystic fibrosis, including carrier frequencies, mutation detection rate, treatment costs, and parameters characterizing carrier screening behavior, such as utilization of preconception screening and prenatal screening, and likelihoods of screening the partner and fetus following a positive test have the largest effects on the results. Figure S3 shows the distribution of incremental costs and number of averted affected childbirths of NGS as compared with genotyping for 1000 simulations with parameter sampling. In 98% of cases, carrier screening by NGS is associated with an increase in the number of averted affected childbirths and a decrease in direct costs. Based on the results of single‐parameter and probabilistic sensitivity analyses, we determined that variations in model parameters do not change the conclusions of the study.

## Discussion

By examining carrier screening using NGS and traditional genotyping technology (which typically looks for only a limited number of mutations per disease) and by capturing the important decisions and outcomes related to preconception and prenatal carrier screening, this study demonstrated that, despite uncertainties in the model inputs, NGS‐based carrier screening is likely to be more cost‐effective than traditional genotyping, averting more affected births, creating more LYs gained, and reducing annual and lifetime treatment costs. From a clinical perspective, carrier screening by NGS averted 21 additional affected births as compared with genotyping, and substantially increased the LYs gained by carrier screening (8636 vs. 7918). From a cost‐effectiveness perspective, while NGS screening offered the greatest benefit in clinical outcomes, it did so at a lower overall healthcare cost as compared with genotyping.

This analysis was based on a number of important but generally conservative assumptions. First, the model accounts for the fact that some couples, when deciding whether to proceed with carrier screening, fetal screening, or an affected pregnancy, might not make the decisions that could prevent the births of children affected by recessive disorders. If we assumed the carrier screening rate and the termination rate of identified affected pregnancies both to be 100%, the NGS strategy would be even more effective, preventing 290 affected births, 28 (44%) more than the genotyping strategy.

Second, the analyses were performed for a population with a race/ethnicity distribution similar to the U.S. population. Our subgroup analysis indicates that, for some subpopulations and screening scenarios, NGS‐based carrier screening could actually result in an overall total savings as compared with no screening. For instance, screening all 14 recessive disorders by NGS in 1,000,000 Ashkenazi Jewish couples resulted in a substantial savings of $421 million in total healthcare costs as compared with no screening.

Third, several model parameters, such as mutation rates and treatment costs of recessive disorders, were inferred from limited data in literature. Whenever assumptions had to be made, we erred on the side of lower cost effectiveness of the NGS strategy. Throughout, sensitivity analyses confirmed that any uncertainties in cost estimates do not change the conclusions of the study.

Finally, our study focused on only the recessive disorders currently recommended for screening by relevant guidelines. We do not opine here as to whether those guidelines should be updated or expanded. (As guidelines do evolve and expand, we would recommend that cost‐effectiveness analyses be performed on such expanded panels.) The benefits of NGS versus smaller mutation sets typically employed by genotyping‐based test panels would be even greater if additional, carefully selected disorders were included, assuming the performance for these tests is also sufficiently high. This conclusion has been independently supported by Lebo and Tonk (2015), who suggested that targeting 64 of the frequent worldwide genetic abnormalities would readily identify the largest proportion of at‐risk couples for affected fetuses. At the other extreme, it should be noted that, while genotyping‐based panels for 100 or more recessive disorders exist, the mutation detection rates of these panels for increasingly rare recessive disorders (with a carrier frequency of 1 in 200 or less) are abysmally low. For rare recessive disorders, the mutation detection rates of genotyping are estimated to be less than 10% for one third of the disorders, and less than 40% for one half of the disorders included in these broader panels of tests (Counsyl, 2010). Assuming that carrier screening for 97 disorders with an average mutation carrier frequency of 1 in 300 were offered to 1,000,000 couples, we found that, for the 35 disorders with detection rates less than 10%, genotyping would yield false‐negative results for at least 385 of the 389 carrier couples, and for the 62 disorders with detection rates less than 50%, there would be false‐negative results for 646 of the 689 carrier couples. In other words, screening for a large number of rare disorders (breadth) with low detection rates (poor depth) provides uninformative results and is largely irrelevant from a medical perspective. This argues that providing high detection rates for the most prevalent genetic disorders via NGS is preferable to providing inferior clinical sensitivity and specificity testing for a wider range of genetic disorders via genotyping and/or using small mutation sets for each disease. (This conclusion is also supported by Lebo and Tonk.)

In summary, carrier screening for recessive disorders using NGS can prevent and inform the majority of affected births, can lead to substantial improvements in health outcomes for the offspring of carriers, and dominates carrier screening by genotyping or by other methods using small mutation sets. These findings offer an evidence‐based justification for a shift in the carrier screening clinical approach from traditional genotyping, covering a wide range of disorders with low accuracy, to NGS, and focusing on the most prevalent disorders with high accuracy.

## Conflict of Interest

Dr. Greger, Ms. Neitzel, and Mr. Rochelle are employees of Good Start Genetics, Inc., and hold stock options in the company. Dr. Azimi, Mr. Schmaus, and Dr. Dinh are employed by Evidera, which provides consulting and other research services to pharmaceutical, device, government, and nongovernment organizations. In their salaried positions, Dr. Azimi, Mr. Schmaus, and Dr. Dinh work with a variety of companies and organizations, and are precluded from receiving payment or honoraria directly from these organizations for services rendered. Evidera received funding from Good Start Genetics, Inc.

## Supporting information


**Appendix S1.** NGS Carrier Screening Benefits, Cost Effectiveness: Details on Model Structure, Outcomes, and Sensitivity Analyses
