# Supplementary material for: Carrier screening by next‐generation sequencing: health benefits and cost effectiveness
Source: Mol Genet Genomic Med. 2016 Jan 29;4(3):292–302. doi: 10.1002/mgg3.204 (PMC4867563; doi:10.1002/mgg3.204)
Supplement: Supplementary file 1 — Appendix S1. NGS Carrier Screening Benefits, Cost Effectiveness: Details on Model Structure, Outcomes, and Sensitivity Analyses [file MGG3-4-292-s001.pdf]

NGS CARRIER SCREENING BENEFITS, COST EFFECTIVENESS: DETAILS ON MODEL STRUCTURE, OUTCOMES, AND SENSITIVITY ANALYSES

SUPPLEMENTARY APPENDIX S1

Table of Contents

A. Enumeration of Outcomes Modeled by the Decision Tree 1
B. Disorder Models 5
C. Carrier Detection Rates 14
D. Lifetime Medical Costs 19
E. Sensitivity Analysis 22
Appendix Figure Legends 24
References 25

A. Enumeration of Outcomes Modeled by the Decision Tree

Tables S1, S2, and S3 enumerate the probabilities associated with different outcomes of pregnancies and their associated costs for each branch of the decision tree described in the manuscript.

Table S1. Enumeration of All Outcomes with Associated Costs for Pregnancies that Utilize Preconception Carrier Screening

Description	Outcome	Cost
Mothers screened	$\sum_{i=1}^{ethnicities} N \cdot D_i \cdot p_{preconception\ screen}$	C_{screen}
Affected children of undetected carrier mothers	$\sum_{i=1}^{ethnicities} N \cdot D_i \cdot p_{preconception\ screen} \cdot p_{carrier,i}^2 \cdot \left(1 - p_{detection,i}\right) \cdot \frac{1}{4}$	$C_{affected}$
Partners screened	$\sum_{i=1}^{ethnicities} N \cdot D_i \cdot p_{preconception\ screen} \cdot p_{carrier,i} \cdot p_{detection,i} \cdot p_{partner\ screen}$	C_{screen}
Affected children of unscreened partners	$\sum_{i=1}^{ethnicities} N \cdot D_i \cdot p_{preconception\ screen} \cdot p_{carrier,i}^2 \cdot p_{detection,i} \cdot \left(1 - p_{partner\ screen}\right) \cdot \frac{1}{4}$	$C_{affected}$
Affected children of undetected carrier partners	$\sum_{i=1}^{ethnicities} N \cdot D_i \cdot p_{preconception\ screen} \cdot p_{carrier,i}^2 \cdot p_{detection,i} \cdot p_{partner\ screen} \cdot \left(1 - p_{detection,i}\right) \cdot \frac{1}{4}$	$C_{affected}$

Description	Outcome	Cost
Couples performing fetal screening	$\left[\sum_{i=1}^{ethnicities} N \cdot D_i \cdot p_{preconception\ screen} \cdot p_{carrier,i}^2 \cdot p_{detection,i}^2 \cdot p_{partner\ screen} \cdot p_{conceive\ at-risk}\right] + N_{previously\ terminated} \cdot p_{conceive\ at-risk}$	$C_{fetalScreen}$
Affected children of carrier couples conceiving at-risk and not terminating	$\left[\sum_{i=1}^{ethnicities} N \cdot D_i \cdot p_{preconception\ screen} \cdot p_{carrier,i}^2 \cdot p_{detection,i}^2 \cdot p_{partner\ screen} \cdot p_{conceive\ at\text{-}risk} \cdot \frac{1}{4} \cdot (1 - p_{terminate})\right] + N_{previously\ terminated} \cdot p_{conceive\ at\text{-}risk} \cdot \frac{1}{4} \cdot (1 - p_{terminate})$	$C_{affected}$
Terminated pregnancies of carrier couples conceiving at-risk	$\left[\sum_{i=1}^{ethnicities} N \cdot D_i \cdot p_{preconception\ screen} \cdot p_{carrier,i}^2 \cdot p_{detection,i}^2 \cdot p_{partner\ screen} \cdot p_{conceive\ at\text{-}risk} \cdot \frac{1}{4} \cdot p_{terminate}\right] + N_{previously\ terminated} \cdot \frac{1}{4} \cdot p_{terminate}$	$C_{terminate}$
Carrier couples pursuing ART	$\left[\sum_{i=1}^{ethnicities} N \cdot D_i \cdot p_{preconception\ screen} \cdot p_{carrier,i}^2 \cdot p_{detection,i}^2 \cdot p_{partner\ screen} \cdot p_{ART}\right] + N_{previously\ terminated} \cdot p_{ART}$	C_{ART}

N, number of partners in population; $\boldsymbol{D_i}$, fraction of couples of a particular ethnicity; $\boldsymbol{p_{preconception\ screen}}$, probability of a couple opting for pre-conception screening; $\boldsymbol{p_{carrier}}$, probability of being a carrier (ethnicity-specific); $\boldsymbol{p_{detection}}$, probability of mutation detection (ethnicity-specific); $\boldsymbol{p_{partner\ screen}}$, probability of partner availability and willingness to screen; $\boldsymbol{p_{conceive\ at\text{-}risk}}$, probability of a couple choosing to conceive at-risk and screen the fetus; $\boldsymbol{p_{terminate}}$, probability that an affected fetus is terminated; $\boldsymbol{p_{art}}$, probability that a carrier couple chooses to undergo ART; $\boldsymbol{N_{previously\ terminated}}$, number of couples that previously terminated an affected fetus and know their carrier status (combination of terminated pregnancies as a result of pre-conception and prenatal carrier screening).

Table S2. Enumeration of All Outcomes with Associated Costs for Pregnancies that Utilize Prenatal Carrier Screening

Description	Outcome	Cost
Mothers screened	$\sum_{i=1}^{ethnicities} N \cdot D_i \cdot p_{prenatal\ screen}$	C_{screen}
Affected children of undetected carrier mothers	$\sum_{i=1}^{ethnicities} N \cdot D_i \cdot p_{prenatal\ screen} \cdot p_{carrier,i}^2 \cdot \left(1 - p_{detection,i}\right) \cdot 1/4$	$C_{affected}$
Partners screened	$\sum_{i=1}^{ethnicities} N \cdot D_i \cdot p_{prenatal\ screen} \cdot p_{carrier,i} \cdot p_{detection,i} \cdot p_{partner\ screen}$	C_{screen}
Affected children of unscreened partners	$\sum_{i=1}^{ethnicities} N \cdot D_i \cdot p_{prenatal\ screen} \cdot p_{carrier,i}^2 \cdot p_{detection,i} \cdot \left(1 - p_{partner\ screen}\right) \cdot 1/4$	$C_{affected}$
Affected children of undetected carrier partners	$\sum_{i=1}^{ethnicities} N \cdot D_i \cdot p_{prenatal\ screen} \cdot p_{carrier,i}^2 \cdot p_{detection,i} \cdot p_{partner\ screen} \cdot \left(1 - p_{detection,i}\right) \cdot 1/4$	$C_{affected}$
Affected children of carrier couples not performing fetal screening	$\sum_{i=1}^{ethnicities} N \cdot D_i \cdot p_{prenatal\ screen} \cdot p_{carrier,i}^2 \cdot p_{detection,i}^2 \cdot p_{partner\ screen} \cdot \left(1 - p_{fetal\ screen}\right) \cdot 1/4$	$C_{affected}$
Couples performing fetal screening	$\sum_{i=1}^{ethnicities} N \cdot D_i \cdot p_{prenatal\ screen} \cdot p_{carrier,i}^2 \cdot p_{detection,i}^2 \cdot p_{partner\ screen} \cdot p_{fetal\ screen}$	$C_{fetalScreen}$
Terminated pregnancies of carrier couples after fetal screening	$\sum_{i=1}^{ethnicities} N \cdot D_i \cdot p_{prenatal\ screen} \cdot p_{carrier,i}^2 \cdot p_{detection,i}^2 \cdot p_{partner\ screen} \cdot p_{fetal\ screen} \cdot 1/4 \cdot p_{terminate}$	$C_{terminate}$
Affected children of carrier couples continuing pregnancy after positive fetal screen	$\sum_{i=1}^{ethnicities} N \cdot D_i \cdot p_{prenatal\ screen} \cdot p_{carrier,i}^2 \cdot p_{detection,i}^2 \cdot p_{partner\ screen} \cdot p_{fetal\ screen} \cdot 1/4 \cdot \left(1 - p_{terminate}\right)$	$C_{affected}$

N, number of partners in population; D_i, fraction of couples of a particular ethnicity; $p_{prenatal\ screen}$, probability of a couple not opting for pre-conception screening; $p_{carrier}$, probability of being a carrier (ethnicity-specific); $p_{detection}$, probability of mutation detection (ethnicity-specific); $p_{partner\ screen}$, probability of partner availability and willingness to screen; $p_{fetal\ screen}$, probability of a carrier couple choosing to screen the fetus; $p_{terminate}$, probability that an affected

fetus is terminated. Note that couples who terminate after pre-conception or prenatal carrier screening are considered to undergo only a single additional round of reproductive decision making. This assumption sufficiently captures the costs associated with loss replacement.

Table S3. Enumeration of All Outcomes with Associated Costs for Pregnancies that Do Not Utilize Any Genetic Screening

Description	Outcome	Cost
Affected children of carrier couples not screened	$\sum_{i=1}^{ethnicities} N \cdot D_i \cdot \left(1 - p_{preconcetption\ screen} - p_{prenatal\ screen}\right) \cdot p_{carrier,i}^2 \cdot 1/4$	$C_{affected}$

N, number of partners in population; D_i, fraction of couples of a particular ethnicity; $p_{preconception\ screen}$, probability of a couple opting for pre-conception screening; $p_{prenatal\ screen}$, probability of a couple not opting for pre-conception screening; $p_{carrier}$, probability of being a carrier (ethnicity-specific).

B. Disorder Models

Brief Descriptions of Genetic Disorders Evaluated

Bloom's Syndrome

Bloom's syndrome is a disorder characterized by short stature, sun-sensitive skin lesions on the nose and cheeks, increased susceptibility to infections, and greatly increased risk of cancer. Other commonly associated conditions include diabetes, lung disease, and infertility. Some people with Bloom's syndrome show limitations in intellectual abilities, while others have normal intelligence.

Canavan Disease

Canavan disease is a neurodegenerative disorder and one of the most common degenerative cerebral disorders in infancy. Children with the disorder appear to have normal development until a few months of age when symptoms usually appear. Symptoms include: delayed motor development, feeding difficulties, enlarged head circumference, and poor muscle tone. Paralysis, blindness, or hearing loss may also occur. Life expectancy is reduced, with some individuals dying in the first decade and others living into their teens or beyond.

Cystic Fibrosis

Cystic fibrosis is a disorder that causes the body to produce abnormally thick mucus in the lungs, and results in chronic respiratory, digestive, and growth problems, as well as male infertility. The symptoms and severity of cystic fibrosis range from mild to severe, depending on the specific gene mutations involved. In classic cystic fibrosis, symptoms begin in early childhood, and the typical life expectancy is into the late 30s.

Dihydrolipoamide Dehydrogenase Deficiency

Dihydrolipoamide dehydrogenase deficiency (DLD), also known as maple syrup urine disease type III, is a metabolic disorder caused by an enzyme deficiency that results in accumulation of certain nutrients, called amino acids, in the brain and other organs. There are multiple forms of DLD, with various levels of severity and ages of onset. The most severe form of DLD has onset in early infancy, at which time affected individuals experience poor feeding, low muscle tone, frequent episodes of vomiting, lethargy, and developmental delay. If untreated, DLD can lead to seizures, coma, blindness, and death.

Familial Dysautonomia

Familial dysautonomia is a disorder of the nervous system that affects the development and survival of certain cells in the nervous systems. Severity of symptoms varies greatly between individuals, but some common symptoms include: poor muscle tone in infancy, vomiting episodes, recurrent pneumonia, inability to process or respond to pain, inability to regulate temperature, renal dysfunction, and cardiovascular instability. Life expectancy is decreased and there is no cure.

Familial Hyperinsulinism

Familial hyperinsulinism is a disorder causing overproduction of insulin, which leads to low blood sugar, also known as hypoglycemia. Familial hyperinsulinism is most commonly caused by mutations in the *ABCC8* gene. Individuals with familial hyperinsulinism generally show symptoms after birth such as lethargy, irritability, poor sleeping, low muscle tone, and feeding problems. Repeated episodes of hypoglycemia increase the risk for serious complications including seizures, breathing problems, brain damage, and even death. Age of onset and severity varies.

Fanconi Anemia Group C

Fanconi anemia group C is a disorder of the blood that results in short stature, bone marrow failure, and predisposition to cancers, especially leukemia. Other symptoms include abnormalities of the heart, kidney, or skeletal system, as well as learning disabilities or mental retardation. The average lifespan for people who have Fanconi anemia is between 20 and 30 years, with the common causes of death being bone marrow failure or cancer.

Glycogen Storage Disease Type 1A

Glycogen storage disease type 1a is a disorder caused by an enzyme deficiency that results in the buildup of a complex sugar called glycogen in the body's cells. Glycogen accumulation in the body's organs and tissues leads to an onset of symptoms in the first few months of life including: low blood sugar, irritability, enlarged liver, seizures, and respiratory problems. Long-term complications of untreated glycogen storage disease type 1a include: short stature, bleeding problems, kidney disease, and brain damage.

Maple Syrup Urine Disease Type 1A/B

Maple syrup urine disease (MSUD) is a metabolic disorder in which the body cannot process certain nutrients called amino acids. MSUD is named for the characteristic maple syrup smell of the urine and ear wax in affected individuals. The build-up of amino acids in the body is toxic and causes feeding problems,

vomiting, and irritability. If untreated, maple syrup urine disease type 1A/1B may result in seizures, mental retardation, coma, and death.

Mucolipidosis Type IV

Mucolipidosis type IV is a severe neurological disorder. Onset of symptoms generally occurs by the end of the first year of life. Affected individuals often do not develop speech and are unable to walk independently. Vision is normal at birth but deteriorates throughout the first decade of life and most individuals are blind by the early teenage years. Individuals with mucolipidosis type IV typically live into adulthood but have reduced life expectancy.

Niemann-Pick Type A and B

Niemann-Pick disease refers to a group of metabolic disorders caused by the deficiency of a specific enzyme whose role is to break down fatty substances in our body, called lipids. Without this enzyme functioning properly, harmful quantities of lipids can accumulate in the liver, lungs, bone marrow, and brain, eventually causing cell death and the malfunction of major organ systems. There are multiple types of Niemann-Pick disease. Type A is the most severe form, with onset in early infancy. Symptoms of Niemann-Pick type A include enlarged liver and spleen, growth deficiency, progressive loss of muscle tone, feeding and swallowing difficulties, and profound brain damage by six months of age. Average life expectancy is 2–3 years of age. Type B is a milder form of the disease, with no neurologic involvement. Symptoms of Niemann-Pick type B include enlarged liver and spleen, growth retardation, respiratory problems and frequent lung infections. Cardiac disease may also occur. Life expectancy is reduced, though survival into adulthood is common.

Tay-Sachs Disease

Tay-Sachs disease is a neurodegenerative disorder caused by a deficiency of an enzyme called hexosaminidase A, or HEXA. Lack of this enzyme causes rapid and progressive deterioration of the brain and nervous system. Children with Tay-Sachs disease are generally healthy until 3–6 months of age, at which point they begin to lose developmental skills. Over time, these children experience progressive weakness, blindness, seizures, and unresponsiveness. Death typically occurs by age 6. There is also a late-onset form of Tay-Sachs disease with symptoms beginning later in life; however, this form is rare.

Usher Syndrome Type IF

Usher syndrome type IF is a disorder which causes deafness and progressive vision loss. Individuals with Usher syndrome type IF are born with profound bilateral hearing loss. Without early intervention, these individuals rarely develop speech. Because of abnormal inner ear function, which is involved in balance, affected individuals typically begin walking later than usual and children often appear clumsy. Additionally,

these individuals have progressive loss of sight beginning in adolescence. Initial signs of vision loss include night blindness and a gradual loss of peripheral vision until only the central vision remains (i.e., "tunnel vision"). Cataracts are common and may reduce central vision to perception of light and dark only. Usher syndrome type IF does not affect intelligence or cause any other health problems. Life expectancy is normal.

Usher Syndrome Type III

Usher syndrome type III is a disorder that causes progressive loss of hearing and vision. Children with Usher syndrome type III have normal hearing and vision at birth. Typically, hearing loss begins during late childhood or adolescence and vision loss begins around the time of puberty. Affected individuals will learn to speak and read normally before their hearing and vision decline. Most people are legally blind and profoundly deaf by middle age. People with Usher syndrome type III may also experience difficulties with balance due to inner ear problems. Usher syndrome does not affect intelligence or cause other health problems. Life-expectancy is normal. There is no cure for Usher syndrome type III.

Mutation Carrier Rates

Cystic Fibrosis

Mutation carrier rates for cystic fibrosis in different ethnicities are well characterized in literature. For the current model, we used data provided by the American Congress of Obstetricians and Gynecologists (ACOG) (Table S4). These figures are largely in agreement with those published by the American College of Medical Genetics (ACMG) in 2006.

Table S4. Mutation Carrier Rate by Ethnicity for Cystic Fibrosis

Ethnicity	Mutation Carrier Rate	Source
Caucasian	1/25	(American Congress of Obstetricians and Gynecologists, 2011, Amos et al., 2006)
Hispanic	1/58	(American Congress of Obstetricians and Gynecologists, 2011, Amos et al., 2006)
African American	1/61	(American Congress of Obstetricians and Gynecologists, 2011, Amos et al., 2006)
Asian	1/94	(American Congress of Obstetricians and Gynecologists, 2011, Amos et al., 2006)
Ashkenazi Jewish	1/24	(American Congress of Obstetricians and Gynecologists, 2011)

Other Disorders

The genetic disorders in the model are most prevalent in the Ashkenazi Jewish population, and are relatively rare in most other populations. Therefore, we estimate two mutation carrier rates for each disorder, namely, an Ashkenazi Jewish mutation carrier rate for individuals of Ashkenazi Jewish descent, and an average pan-ethnic rate for other ethnicities. For several disorders, the pan-ethnic carrier rates were not found in the literature; in these cases, estimates based on disease incidence or other existing knowledge were made. We also took advantage of clinical data collected by Good Start Genetics (GSG), and consulted experts on the disorders.

Table S5. Mutation Carrier Rate for the Ashkenazi Jewish Population by Disorder

Disorder	Mutation Carrier Rate		Source	Notes
	Ashkenazi Jewish	Pan-Ethnic		
Bloom's Syndrome	1/134	1/800	(Wang et al., 2013, Hallam et al., 2014, Scott et al., 2010)	As of 2009, 265 cases of Bloom's syndrome had been documented in the Bloom's Syndrome Registry, with 99 cases documented from the United States (US), 43 Jewish and 56 Non-Jewish. Using an AJ carrier frequency of 1/134, and assuming that 2% of the total US population are individuals of AJ descent, we estimate a pan-ethnic carrier rate in the US of approximately 1 in 800. This estimate is consistent with the rate observed in a recent multi-ethnic study (Hallam et al., 2014).
Canavan Disease	1/55	1/300	(Hallam et al., 2014, Scott et al., 2010, Strom et al., 2004)	The pan-ethnic rate is based on data from Hallam et al. (Hallam et al., 2014).

Disorder	Mutation Carrier Rate		Source	Notes
	Ashkenazi Jewish	Pan-Ethnic		
Dihydrolipoamide Dehydrogenase Deficiency	1/107	1/1000	(Counsyl, 2014, Hallam et al., 2014)	Using data from Hallam et al. (Hallam et al., 2014), which reported 4 carriers (2 are of AJ descent) in a screened population of 2015, we estimated the pan-ethnic rate for Dihydrolipoamide Dehydrogenase Deficiency (DDD) to be approximately 1 in 1000, which is consistent with the fact that the majority of cases coming from families of AJ background.
Familial Dysautonomia	1/31	1/300	(Hallam et al., 2014, Scott et al., 2010)	The pan-ethnic rate is based on data from Hallam et al. (Hallam et al., 2014).
Familial Hyperinsulinism	1/68	1/112	(Hallam et al., 2014, Scott et al., 2010)	The pan-ethnic rate is based on data from Hallam et al. (Hallam et al., 2014).
Fanconi Anemia Group C	1/100	1/300	(Terfve and Saez-Rodriguez, 2012, Hallam et al., 2014, Scott et al., 2010)	The pan-ethnic rate is based on data from Hallam et al. (Hallam et al., 2014).
Glycogen Storage Disease Type 1a	1/64	1/177	(Chou et al., 2002, Scott et al., 2010)	
Maple Syrup Urine Disease Type 1	1/97	1/215	(Strauss et al., 2006 Jan 30 [Updated 2013 May 9])	
Mucolipidosis Type IV	1/89	1/300	(Scott et al., 2010)	The pan-ethnic rate is based on data from Hallam et al. (Hallam et al., 2014).

Disorder	Mutation Carrier Rate		Source	Notes
	Ashkenazi Jewish	Pan-Ethnic		
Niemann-Pick Disease Type A	1/115	1/1000	(Scott et al., 2010, Simonaro et al., 2002)	Niemann-Pick type A is predominantly associated with the Ashkenazi Jewish population.
Niemann-Pick Disease Type B	1/1000	1/250	(Genetics Home Reference, 2014, Scott et al., 2010, Simonaro et al., 2002)	Niemann-Pick type B is predominantly associated with the non-Jewish populations.
Tay-Sachs Disease	1/27	1/300	(Scott et al., 2010, Strom et al., 2004, Kaback and Desnick, 1999 Mar 11 [Updated 2011 Aug 11], Warren et al., 2005)	Genetics experts estimated carrier rate of Tay-Sachs Disease in pan-ethnic population to be 1 in 300, which is consistent with other estimates in literature (Warren et al., 2005).
Usher Syndrome Type IF	1/147	1/300	(Scott et al., 2010)	The pan-ethnic rate is based on data from Hallam et al. (Hallam et al., 2014).
Usher Syndrome Type III	1/120	1/500	(Scott et al., 2010)	The pan-ethnic rate is based on data from Hallam et al. (Hallam et al., 2014).

Life Expectancy

For births unaffected by any genetic disorder, an average life expectancy of 78.5 was used, derived from United States Life Tables from the Centers for Disease Control and Prevention (CDC) (Arias, 2014). For other disorders, estimates were drawn from published data in literature.

Based on discussions with clinical experts, we assumed that the following disorders do not affect life expectancy: maple syrup urine disease (Strauss et al., 2006 Jan 30 [Updated 2013 May 9]) and both Usher syndrome Type IF and Type III (Keats and Lentz, 1999 Dec 10 [Updated 2013 Jun 20]).

To estimate life expectancy for Canavan disease and familial hyperinsulinism, we used United States mortality data by age and ICD-10 code, from 1999 through 2007. The data was obtained from the website www.icd10data.com, and was sourced from the CDC (Ciaccio et al., 2010). The disorders we considered did not have their own ICD-10 codes with mortality data available, so we necessarily assumed life expectancy was similar to the other disorders with which they were grouped. Canavan disease is assigned the ICD-10 code E75.2, "Other sphingolipidosis," and had an average age at death of 21.0. This was consistent with descriptions of life expectancy found in the literature (Matalon and Michals-Matalon, 1999 Sep 16 [Updated 2011 Aug 11]). Familial hyperinsulinism is assigned the ICD-10 code E16.1, "Other hypoglycemia," and had an average age at death of 60.0 years.

Many disorders were characterized in the literature as having significant heterogeneity with respect to survival, with some individuals dying in infancy, and others surviving into adulthood. These disorders included dihydrolipoamide dehydrogenase deficiency (Shaag et al., 1999), Fanconi anemia group C (Terfve and Saez-Rodriguez, 2012, Kutler et al., 2003), glycogen storage disease type 1A (Rake et al., 2002), mucolipidosis type IV (Badidi et al., 2003), and Niemann-Pick type B. For the sake of simplicity, we assumed a life expectancy of 30 years.

Table S6. Life Expectancy by Disorder

Disorder	Life Expectancy	Source
Bloom's Syndrome	26.0	(Wang et al., 2013)
Canavan Disease	21.0	(Ciaccio et al., 2010, Matalon and Michals-Matalon, 1999 Sep 16 [Updated 2011 Aug 11])
Cystic Fibrosis	41.0	(D'Haeseleer et al., 2000)
Dihydrolipoamide Dehydrogenase Deficiency	30.0	(Shaag et al., 1999), *Assumption*
Familial Dysautonomia	15.0	(Khan et al., 2011)
Familial Hyperinsulinism	60.0	(Ciaccio et al., 2010)
Fanconi Anemia Group C	30.0	(Terfve and Saez-Rodriguez, 2012, Kutler et al., 2003)

Disorder	Life Expectancy	Source
Glycogen Storage Disease Type 1a	30.0	(Rake et al., 2002), *Assumption*
Maple Syrup Urine Disease Type 1	78.5	(Arias, 2014)
Mucolipidosis Type IV	30.0	(Badidi et al., 2003)*Assumption*
Niemann-Pick Disease Type A	3.0	(Genetics Home Reference, 2014)
Niemann-Pick Disease Type B	30.0	(Schuchman, 2007), *Assumption*
Tay-Sachs Disease	3.0	(Kaback and Desnick, 1999 Mar 11 [Updated 2011 Aug 11])
Usher Syndrome Type IF	78.5	(Center for Jewish Genetics, 2014)
Usher Syndrome Type III	78.5	(Center for Jewish Genetics, 2014)

C. Carrier Detection Rates

Detection rates describe the percentage of disease-causing mutations that a given test will identify. Detection rates for carriers of common mutations in target populations are well established. Numerous additional mutations, although less frequent or very rare individually, often account for a sizeable fraction of carriers in aggregate, particularly in non-target populations. Accurately calculating their (individual) contribution to detection rates would require large data sets that currently are not available; when derived from small studies, their allele frequencies are routinely overestimated and thus inflate the detection rate.

As a result, depending on the particular study chosen for the calculation, the determined detection rate for the same panel will vary considerably. (Because there is no standardized method for calculating detection rates, these figures may differ significantly from laboratory to laboratory.) Next-generation sequencing allows for detection of more mutations than traditional genotyping-based carrier screens, while still detecting so-called common mutations. As a result, NGS is expected to yield higher detection rates than older, traditional approaches. Given the lack of literature to support the actual detection rate NGS would provide, we followed the algorithm below for estimating detection rates.

- Conservative base-line detection rates for traditional genotyping were drawn from published literature. Only the largest studies were taken into account.
- Of the remaining percentages to be detected, a fraction is due to novel truncating mutations. This fraction varies by gene and disorder and was calculated using the fraction of truncating mutations among all known pathogenic mutations for each gene. The percentage of remaining mutations (detection rate) due to truncating mutations is calculated by multiplying the total remaining detection by the percentage of mutations that are truncating in that gene, for individuals of each ethnicity. We assume a 2% false negative rate (i.e., 98% detection) of these novel truncating mutations by NGS.
- The additional percentage detection due to novel truncating mutations is added to the baseline detection rates to yield final NGS detection rates.
- The contribution of rare, known pathogenic mutations to the detection rate is not specifically taken into account. Since virtually all of those mutations are truncating, their impact is considered to be subsumed under the novel, truncating category.

Therefore, we estimate the next-generation sequencing detection rate using the following formula:

$$P_{NGS} = P_{Genotyping} + 0.98 \times P_{TruncationRate} \times (1 - P_{Genotyping})$$

where P_{NGS} is the detection rate for next-generation sequencing, and $P_{TruncationRate}$ is the truncation rate.

The truncation rate is estimated based on Hallam et al. (Hallam et al., 2014) and expert opinion. The approach described above provides a conservative way to estimate the accuracy of NGS and appears to be consistent with existing data. To estimate mutation detection rates, we used data provided by Good Start Genetics. Table S7 compares the number of mutation carriers detected by NGS in this population against those would be detected by different traditional genotyping assays. Of the 3,093 carriers detected among 71,070 patients screened in the pattern of tests ordered by individual caregivers in the clinical setting, 11.0%–25.8% would have been missed by other major laboratories using traditional genotyping.

We verified that the model is consistent with data published in literature. For instance, for cystic fibrosis, Hallam et al.(Hallam et al., 2014) reported that NGS identified a total of 335 mutations; among these were 12 included in limited panels, and 7 unique to NGS, including one novel mutation, c.1526delG, found in an Asian patient. This falls within the range of improvements predicted by the model. For Canavan disease, in the non-AJ population, NGS panel detected 12 mutations; among these 2 are unique to NGS, which is consistent with an increase in mutation detection rate in the non-AJ population from 53% to 64%, as predicted by the model. The improvements in detection rate for several disorders are remarkable. For instance, the mutation detection rates for Usher syndrome type IF increase from 64% and 10% to 89% and 72% for AJ and pan-ethnic populations, respectively, which is consistent with the fact that two out of three mutations detected are unique to NGS.

Table S7. Comparison of Mutation Detection of NGS vs. Genotyping Assays*

DISEASE	GENE	OMIM Number	Quest	LabCorp	Counsyl	GenPath	Progenity	Recombine	GSG TOTAL
Familial Hyperinsulinism	*ABCC8*	#256450	0	40	41	41	41	41	**62**
Canavan Disease	*ASPA*	#271900	73	73	73	73	73	74	**87**
Maple Syrup Urine Disease 1A	*BCKDHA*	#248600	0	3	0	3	3	7	**24**
Maple Syrup Urine Disease 1B	*BCKDHB*	#248600	47	47	47	47	47	47	**77**
Bloom Syndrome	*BLM*	#210900	22	22	22	34	22	33	**63**
Cystic Fibrosis	*CFTR*	#219700	1735	1928	1913	2009	1963	1927	**2056**
Usher Syndrome Type III	*CLRN*	#276902	0	26	26	28	26	28	**43**
Dihydrolipoamide Dehydrogenase Deficiency	*DLD*	#246900	0	31	31	31	31	31	**35**
Fanconi Anemia Group C	*FANCC*	#227645	42	42	45	47	48	54	**81**
Glycogen Storage Disease Type IA	*G6PC*	#232200	65	92	85	89	92	93	**104**
Tay-Sachs Disease DNA	*HEXA*	#272800	150	150	157	166	152	170	**195**
Familial Dysautonomia	*IKBKAP*	#223900	76	76	76	76	76	76	**98**
Mucolipidosis Type IV	*MCOLN1*	#252650	36	36	36	36	36	36	**46**
Usher Syndrome Type 1F	*PCDH15*	#602083	0	21	21	22	21	21	**47**
Niemann Pick Disease Type A/B	*SMPD1*	#257200, #607616	50	50	50	51	51	51	**75**
	**Total**		2296	2637	2623	2753	2682	2689	**3093**
	**Missed**		**797**	**456**	**470**	**340**	**411**	**404**	
	**% Missed**		**25.8%**	**14.7%**	**15.2%**	**11.0%**	**13.3%**	**13.1%**	

* Data is derived from a clinical database of 71,070 patients.

Abbreviation: OMIM®=*Online Mendelian Inheritance in Man;* GSG=Good Start Genetics

Table S8. Mutation Detection Rates – Ashkenazi Jewish Ethnicity

Disorder	Traditional Genotyping	Next Generation Sequencing	Source
Bloom's Syndrome	0.9700	0.9888	(Counsyl, 2010)
Canavan Disease	0.9880	0.9904	(Counsyl, 2010, Kaul et al., 1994)
Cystic Fibrosis	0.9540	0.9698	(Counsyl, 2010, Heim et al., 2001)
Dihydrolipoamide Dehydrogenase Deficiency	0.9900	0.9915	(Counsyl, 2010, Shaag et al., 1999)
Familial Dysautonomia	0.9950	0.9990	(Counsyl, 2010, Lehavi et al., 2003)
Familial Hyperinsulinism	0.8800	0.9212	(Counsyl, 2010, Nestorowicz et al., 1996)
Fanconi Anemia Group C	0.9900	0.9953	(Counsyl, 2010, Scott et al., 2010)
Glycogen Storage Disease Type 1a	0.9710	0.9795	(Counsyl, 2010, Ekstein et al., 2004, Janecke et al., 2001)
Maple Syrup Urine Disease Type 1	0.9500	0.9701	(Scott et al., 2010)
Mucolipidosis Type IV	0.9600	0.9761	(Counsyl, 2010, Edelmann et al., 2002)
Niemann-Pick Disease Type A/B	0.9700	0.9776	(Counsyl, 2010, Schuchman and Miranda, 1997)
Tay-Sachs Disease	0.9420	0.9613	(Kaback and Desnick, 1999 Mar 11 [Updated 2011 Aug 11])
Usher Syndrome Type IF	0.6400	0.8870	(Ben-Yosef et al., 2003)
Usher Syndrome Type III	0.9900	0.9949	(Ness et al., 2003)

Table S9. Mutation Detection Rates – Other Ethnicities

**Disorder**	**Traditional Genotyping**	**Next Generation Sequencing**	**Source**
Bloom's Syndrome	0.1000	0.6645	(Counsyl, 2010)
Canavan Disease	0.5337	0.6251	(Counsyl, 2010, Kaul et al., 1994)
Cystic Fibrosis (Caucasian)	0.9164	0.9451	(Counsyl, 2010, Palomaki et al., 2002)
Cystic Fibrosis (Hispanic)	0.7500	0.8358	(Counsyl, 2010, Wong et al., 2001)
Cystic Fibrosis (African American)	0.8100	0.8752	(Counsyl, 2010, Heim et al., 2001)
Cystic Fibrosis (Asian)	0.4893	0.6645	(Counsyl, 2010)
Dihydrolipoamide Dehydrogenase Deficiency	0.1000	0.2323	(Counsyl, 2010)
Familial Dysautonomia	0.1000	0.1500	(Counsyl, 2010)
Familial Hyperinsulinism	0.1000	0.4087	(Counsyl, 2010)
Fanconi Anemia Group C	0.5980	0.8107	(Counsyl, 2010)
Glycogen Storage Disease Type 1A (Asian)	0.8860	0.9195	(Janecke et al., 2001, Ki et al., 2004)
Glycogen Storage Disease Type 1A (Other)	0.5460	0.6795	(Janecke et al., 2001, Ki et al., 2004)
Maple Syrup Urine Disease Type 1 (Caucasian)	0.4005	0.6414	(Counsyl, 2010)
Maple Syrup Urine Disease Type 1 (Other)	0.1000	0.4616	(Counsyl, 2010)
Mucolipidosis Type IV	0.1000	0.4616	(Counsyl, 2010)
Niemann-Pick Disease Type A/B	0.1000	0.3293	(Counsyl, 2010)
Tay-Sachs Disease	0.5200	0.6799	(Park et al., 2010)
Usher Syndrome Type IF	0.1000	0.7174	(Counsyl, 2010)
Usher Syndrome Type III	0.1000	0.5410	(Counsyl, 2010)

D. Lifetime Medical Costs

When possible, lifetime medical costs were derived from information in the literature. As each disorder is rare, estimates were not always available. In those cases, available information was leveraged, and disorder experts were consulted.

Bloom's syndrome is a very rare disorder, with only 265 documented cases in the Bloom's syndrome registry. People with Bloom's syndrome have an increased risk of cancer. Cancers can arise early in life, and thus necessitate frequent cancer screening. Those with Bloom's syndrome are also at increased risk for diabetes, chronic obstructive pulmonary disorder, and recurrent infections of the upper respiratory tract, ears, and lungs. There are no published resources estimating the cost of caring for individuals with Bloom's syndrome. Therefore, after consultation with disorder experts, and given a similar disease course, we elected to use the lifetime cost of familial dysautonomia as a proxy cost for Bloom's syndrome.

For determining the lifetime cost of Canavan disease, we use the average yearly per-patient cost for pediatric leukodystrophy patients, as described in Bonkowsky et al. (Bonkowsky et al., 2010). They found the average per-patient cost from 1999 through 2007 was $22,579. We used an inflation factor of 1.47, representing medical inflation from 2003 (the midpoint year of the study) to 2014. Using an average life expectancy of 21 years, we estimate total lifetime cost to be $527,000.

Estimates for the average lifetime medical costs for CF patients vary widely in the literature. In a systematic literature review of CF studies published between 1990 and 2006, lifetime costs ranged from $329,388 to $1,251,074 (Radhakrishnan et al., 2008). Many previous CF studies have used the National Institute of Health (NIH) consensus estimate of $800,000, published in 1997 (National Institutes of Health (NIH), 1997 (April 14-16)). Updating the NIH estimate using medical care inflation to 2013 dollars gives an estimate of $1.47 million. Since 1997, the life expectancy of CF patients has greatly improved, from a median survival age near 30 in 1997 to 41.1 in 2012 (D'Haeseleer et al., 2000). Costs of medical care have also increased. For our analysis, we pooled the estimates of the cost of average annual medical care from Briesacher et al. (2011) (Briesacher et al., 2011) and Ouyang et al. (2009) (Ouyang et al., 2009). Both analyses separate patients by age, with older patients having higher average costs. Costs were updated to 2014 dollars. We define our estimate of the lifetime cost as follows:

$$\text{LifetimeCost} = \sum_{\text{age}=1}^{\text{AverageAge}} \frac{\text{Cost(age)}}{(1+r)^{\text{age}-1}}$$

where r is a 3% discount rate. Using a life expectancy of 41 years, the lifetime cost of medical care is estimated at being $1,319,000.

Table S10. Average Cost of Annual Medical Care by Age Range for Cystic Fibrosis

	**Ages 0–10**	**Ages 11–29**	**Ages 30+**
(Ouyang et al., 2009)	$43,928 (N=573)	$67,236 (N=1157)	$69,561 (N=473)
(Briesacher et al., 2011)	$39,633 (N=1013)	$56,293 (N=1440)	$70,111 (N=819)
Pooled	$41,186	$61,169	$69,909

For dihydrolipoamide dehydrogenase deficiency, we assumed a similar lifetime cost to maple syrup urine disease.

For the lifetime cost of familial dysautonomia, we used the cost per affected person between ages 2–18 found in Lines (2013) (Lines, 2013 (March)), namely £59,787 in 2006 UK pounds. In 2006, the exchange rate was one UK pound to 1.84 US dollars. Between 2006 and 2014 medical inflation was approximately 30%. Using an average life expectancy of 15 years, we estimate total lifetime cost to be $1,758,000.

Carroll & Downs. (Carroll and Downs, 2006) report a lifetime cost of $122,515 for maple syrup urine disease, reported in 2004 dollars. Medical inflation from 2004 to 2014 was approximately 40%, so we used a lifetime cost of $172,000.

There are very few estimates of cost in the literature for either Niemann-Pick type A or type B. As Niemann-Pick type A is similar to Tay-Sachs in terms of disease severity and life expectancy, we assume they have similar costs, and assign a cost of $750,000 to Niemann-Pick type A.

Surprisingly, there is very little recent data on the lifetime cost of Tay-Sachs disease. In 1978, Nelson et al. estimated the lifetime cost of Tay-Sachs disease to be between $60,300 and $120,600, in 1971 dollars (Nelson et al., 1978). Since 1971, there has been approximately an 1100% increase in medical costs. Using this inflation factor, we might estimate the current lifetime cost of Tay-Sachs as anywhere from $723,600 to $1,447,200. This estimate seems to fit with recent reporting on the cost of Tay-Sachs (Ramirez, 2006 Aug 24). Therefore, for the purposes of this model, we assume the lifetime cost of Tay-Sachs is $750,000. This is a conservative estimate of the life time cost.

For Usher Syndrome type IF and type III, we use increased medical costs associated with blindness to estimate the lifetime cost of the disorder. Frick et al. estimate yearly excess medical expenditures associated with blindness to be $2,157 in 2004 dollars (Frick et al., 2007). Using an inflation factor of 1.40, representing medical care inflation from 2004 to 2014, we estimate the lifetime cost of disease-induced blindness, and to be approximately $93,000.

No data on medical costs could be found for the following disorders: familial hyperinsulinism, glycogen storage disease type 1A, mucolipidosis type IV, and Niemann-Pick type B. Each one of these disorders has significant heterogeneous expression. After consultation with scientists and disease-area experts, we assigned a conservative lifetime cost of $100,000 to each of these disorders.

Table S11. Lifetime Medical Costs by Disorder

Disorder	Lifetime Cost	Source
Bloom's Syndrome	$1,758,000	Assumption
Canavan Disease	$527,000	(Bonkowsky et al., 2010)
Cystic Fibrosis	$1,319,000	(Briesacher et al., 2011, Ouyang et al., 2009)
Dihydrolipoamide Dehydrogenase Deficiency	$172,000	Assumption
Familial Dysautonomia	$1,758,000	(Lines, 2013 (March))
Familial Hyperinsulinism	$100,000	Assumption
Fanconi Anemia Group C	$750,000	Expert Opinion
Glycogen Storage Disease Type 1a	$100,000	Assumption
Maple Syrup Urine Disease Type 1	$172,000	(Carroll and Downs, 2006)
Mucolipidosis Type IV	$100,000	Assumption
Niemann-Pick Disease Type A	$750,000	Assumption
Niemann-Pick Disease Type B	$100,000	Assumption
Tay-Sachs Disease	$750,000	(Nelson et al., 1978, Ramirez, 2006 Aug 24)
Usher Syndrome Type IF	$93,000	(Frick et al., 2007)
Usher Syndrome Type III	$93,000	(Frick et al., 2007)

E. Sensitivity Analysis

In this section, we reported the results of single-parameter sensitivity analysis and probabilistic sensitivity analysis. We performed the single-parameter sensitivity analysis to identify the model inputs that have the most significant impacts on the predicted outcomes. We varied each model parameter within a range representing plausible upper and lower limits. The ranges of the model parameters are based on literature and summarized in Table 1 of the main article and this Appendix in the table and figures that follow in this section. The effects of varying parameters on averted affected childbirths and total medical costs are summarized in Figure S1 and Figure S2 (top 20 parameters) and Table S12 (all parameters). Overall, there are two groups of parameters that have the most significant impacts on the model predictions:

- Parameters related to clinical and economic aspects of cystic fibrosis, including carrier frequencies, mutation detection rates, treatment costs
- Parameters characterizing carrier screening behavior, such as utilization of pre-conception screening and pre-natal screening and likelihoods of screen partner and fetus following a positive test

For probabilistic sensitivity analysis, we sampled the model parameters from their probable distributions. The results of the probabilistic analysis are summarized in a scatter plot representing the estimated joint density of 1000 resampled estimates of incremental costs and additional averted affected childbirths (Figure S3). For 98% of cases, NGS reduces costs and averts more affected childbirths than genotyping. Based on the results of single-parameter and probabilistic sensitivity analysis, we concluded that the variations in model parameters do not change the conclusion of the study.

Table S12. Sensitivity Analysis

Parameter	Difference in Cost: NGS vs Genotyping<br>With Upper Limit of Parameter Range	Difference in Cost: NGS vs Genotyping<br>With Lower Limit of Parameter Range	Difference in Averted Affected Childbirths: NGS vs Genotyping<br>With Upper Limit of Parameter Range	Difference in Averted Affected Childbirths: NGS vs Genotyping<br>With Lower Limit of Parameter Range
Base	-$12,966,881		21.16	
Screening Modality Utilization: Pre-conception Screening	-$13,568,693	-$12,365,070	22.10	20.22
Screening Modality Utilization: Pre-natal Screening	-$14,055,729	-$11,878,034	23.04	19.28
Pre-Conception Carrier Decision: Conceive and Screen Fetus	-$12,938,130	-$12,995,280	21.16	21.16
Pre-Conception Carrier Decision: ART	-$12,911,274	-$13,022,489	21.16	21.16
Pre-Conception Carrier Decision: Have No Children	-$12,966,881	-$12,966,881	21.16	21.16
Pre-natal Carrier Decision:Screen Fetus	-$15,058,935	-$9,828,801	23.51	17.63
Pre-natal Carrier Decision: Screen Partner	-$15,165,943	-$7,582,972	24.75	12.37
Cost of Carrier Screening	-$7,118,932	-$15,306,061	21.16	21.16
Cost of Terminate Pregnancy	-$12,959,508	-$12,978,611	21.16	21.16
Cost of ART	-$12,894,948	-$12,976,064	21.16	21.16
Cost of Screenfetus	-$12,932,495	-$13,001,267	21.16	21.16
Lifetime Treatment Cost of Cystic Fibrosis	-$21,846,060	-$8,807,619	21.16	21.16
Lifetime Treatment Cost of FanconiAnemiaGroupC	-$13,124,806	-$12,808,957	21.16	21.16
Lifetime Treatment Cost of MucolipidosisTypeIV	-$13,010,935	-$12,944,855	21.16	21.16
Lifetime Treatment Cost of TaySachsDisease	-$13,115,826	-$12,817,937	21.16	21.16
Lifetime Treatment Cost of BloomSyndrome	-$13,026,221	-$12,907,541	21.16	21.16
Lifetime Treatment Cost of CanavanDisease	-$12,996,908	-$12,936,855	21.16	21.16
Lifetime Treatment Cost of NiemannPickDiseaseTypeA	-$12,971,169	-$12,962,594	21.16	21.16
Lifetime Treatment Cost of NiemannPickDiseaseTypeB	-$12,974,306	-$12,959,457	21.16	21.16
Lifetime Treatment Cost of DihydrolipoamideDehydrogenaseDeficiency	-$12,967,281	-$12,966,482	21.16	21.16
Lifetime Treatment Cost of FamilialDysautonomia	-$12,992,424	-$12,941,339	21.16	21.16
Lifetime Treatment Cost of GlycogenStorageDiseaseType1A	-$12,991,092	-$12,942,671	21.16	21.16
Lifetime Treatment Cost of MapleSyrupUrineDisease	-$13,008,748	-$12,925,015	21.16	21.16
Lifetime Treatment Cost of FamilialHyperinsulinsim	-$13,027,428	-$12,906,334	21.16	21.16
Lifetime Treatment Cost of UsherSyndromeType1F	-$12,993,021	-$12,940,741	21.16	21.16
Lifetime Treatment Cost of UsherSyndromeTypeIII	-$12,971,898	-$12,961,865	21.16	21.16
Expected LY Healthy	-$12,966,881	-$12,966,881	21.16	21.16
Expected LY CysticFibrosis	-$12,966,881	-$12,966,881	21.16	21.16
Expected LY FanconiAnemiaGroupC	-$12,966,881	-$12,966,881	21.16	21.16
Expected LY MucolipidosisTypeIV	-$12,966,881	-$12,966,881	21.16	21.16
Expected LY TaySachsDisease	-$12,966,881	-$12,966,881	21.16	21.16
Expected LY BloomSyndrome	-$12,966,881	-$12,966,881	21.16	21.16
Expected LY CanavanDisease	-$12,966,881	-$12,966,881	21.16	21.16
Expected LY NiemannPickDiseaseTypeA	-$12,966,881	-$12,966,881	21.16	21.16
Expected LY NiemannPickDiseaseType B	-$12,966,881	-$12,966,881	21.16	21.16
Expected LY Dihydrolipoamide Dehydrogenase Deficiency	-$12,966,881	-$12,966,881	21.16	21.16
Expected LY Familial Dysautonomia	-$12,966,881	-$12,966,881	21.16	21.16
Expected LY Glycogen Storage Disease Type 1A	-$12,966,881	-$12,966,881	21.16	21.16
Expected LY Maple Syrup Urine Disease	-$12,966,881	-$12,966,881	21.16	21.16
Expected LY Familial Hyperinsulinsim	-$12,966,881	-$12,966,881	21.16	21.16
Expected LY Usher Syndrome Type 1F	-$12,966,881	-$12,966,881	21.16	21.16
Expected LY Usher Syndrome Type III	-$12,966,881	-$12,966,881	21.16	21.16
Carrier Frequency: Cystic Fibrosis	-$6,987,773	-$25,936,113	16.47	31.30
Carrier Frequency: Fanconi Anemia Group C	-$12,861,051	-$13,224,908	20.93	21.65
Carrier Frequency: Mucolipidosis Type IV	-$13,055,974	-$12,824,423	21.00	21.50
Carrier Frequency: Tay Sachs Disease	-$12,856,837	-$13,227,290	20.95	21.62
Carrier Frequency: Bloom Syndrome	-$12,941,914	-$13,050,139	21.11	21.27
Carrier Frequency: Canavan Disease	-$12,951,133	-$13,013,548	21.08	21.34
Carrier Frequency: Niemann Pick Disease Type A	-$12,980,223	-$12,947,600	21.15	21.18
Carrier Frequency: Niemann Pick Disease Type B	-$13,035,514	-$12,856,559	21.05	21.39
Carrier Frequency: Dihydrolipoamide Dehydrogenase Deficiency	-$12,977,493	-$12,949,442	21.16	21.17
Carrier Frequency: Familial Dysautonomia	-$12,944,767	-$13,021,665	21.14	21.21
Carrier Frequency: Glycogen Storage Disease Type 1A	-$13,001,047	-$12,923,196	20.81	21.91
Carrier Frequency: Maple Syrup Urine Disease	-$13,024,734	-$12,896,251	20.81	21.92
Carrier Frequency: Familial Hyperinsulinsim	-$13,131,731	-$12,725,284	20.29	23.04
Carrier Frequency: Usher Syndrome Type 1F	-$13,113,863	-$12,735,903	20.76	22.04
Carrier Frequency: Usher Syndrome Type III	-$13,035,417	-$12,855,341	21.08	21.33
NGS Detection Rate: Cystic Fibrosis	-$17,180,119	-$8,801,208	24.51	17.85
NGS Detection Rate: Fanconi Anemia Group C	-$13,028,853	-$12,913,523	21.35	20.98
NGS Detection Rate: Mucolipidosis Type IV	-$12,855,325	-$13,081,076	21.36	21.00
NGS Detection Rate: Tay Sachs Disease	-$13,030,425	-$12,908,383	21.33	21.00
NGS Detection Rate: Bloom Syndrome	-$13,002,175	-$12,951,962	21.22	21.11
NGS Detection Rate: Canavan Disease	-$12,966,351	-$12,968,515	21.22	21.10
NGS Detection Rate: Niemann Pick Disease Type A	-$12,949,135	-$12,985,530	21.17	21.15
NGS Detection Rate: Niemann Pick Disease Type B	-$12,880,333	-$13,054,956	21.28	21.06
NGS Detection Rate: Dihydrolipoamide Dehydrogenase Deficiency	-$12,953,494	-$12,980,330	21.16	21.16
NGS Detection Rate: Familial Dysautonomia	-$12,977,232	-$12,957,681	21.18	21.15
NGS Detection Rate: Glycogen Storage Disease Type 1A	-$12,911,720	-$13,023,026	21.43	20.90
NGS Detection Rate: Maple Syrup Urine Disease	-$12,881,230	-$13,058,804	21.51	20.85
NGS Detection Rate: Familial Hyperinsulinsim	-$12,758,020	-$13,189,542	22.21	20.29
NGS Detection Rate: Usher Syndrome Type 1F	-$12,785,119	-$13,155,726	21.69	20.73
NGS Detection Rate: Usher Syndrome Type III	-$12,881,400	-$13,053,646	21.26	21.08

Appendix Figure Legends

Figure S1. Single-parameter Sensitivity Analysis: Tornado Diagram of the Results of the Top 20 Parameters that Have the Most Significant Impacts on Total Number of Cases Averted between NGS and Genotyping.

Figure S2. Single-parameter Sensitivity Analysis: Tornado Diagram of the Results of the Top 20 Parameters that Have the Most Significant Impacts on Total Cost Differences between NGS and Genotyping

Figure S3. Probabilistic Sensitivity Analysis of Model Predictions

References

American Congress of Obstetricians and Gynecologists 2011. ACOG Committee Opinion No. 486: Update on carrier screening for cystic fibrosis. *Obstetrics and Gynecology,* 117, 4, 1028-1031.

Amos, J., Feldman, G. L., Grody, W. W., Monaghan, K., Palomaki, G. E., Prior, T. W., Richards, C. S. & Watson, M. S. 2006. *American College of Medical Genetics: Technical standards and guidelines for CFTR mutation testing: 2006 Edition.* [Online]. Bethesda, MD: American College of Medical Genetics. Available: https://www.acmg.net/Pages/ACMG_Activities/stds-2002/cf.htm [Accessed 20 June 2014].

Arias, E. 2014. *United States life tables, 2009. National Vital Statistics Reports; Vol 62, No 7.* [Online]. Hyattsville, MD: National Center for Health Statistics, Centers for Disease Control and Prevention. Available: http://www.cdc.gov/nchs/data/nvsr/nvsr62/nvsr62_07.pdf [Accessed 1 August 2014].

Badidi, E., De Sousa, C., Lang, B. F. & Burger, G. 2003. AnaBench: a Web/CORBA-based workbench for biomolecular sequence analysis. *BMC Bioinformatics,* 4, 63.

Ben-Yosef, T., Ness, S. L., Madeo, A. C., Bar-Lev, A., Wolfman, J. H., Ahmed, Z. M., Desnick, R. J., Willner, J. P., Avraham, K. B., Ostrer, H., Oddoux, C., Griffith, A. J. & Friedman, T. B. 2003. A mutation of PCDH15 among Ashkenazi Jews with the type 1 Usher syndrome. *New England Journal of Medicine,* 348, 17, 1664-1670.

Bonkowsky, J. L., Nelson, C., Kingston, J. L., Filloux, F. M., Mundorff, M. B. & Srivastava, R. 2010. The burden of inherited leukodystrophies in children. *Neurology,* 75, 8, 718-725.

Briesacher, B. A., Quittner, A. L., Fouayzi, H., Zhang, J. & Swensen, A. 2011. Nationwide trends in the medical care costs of privately insured patients with cystic fibrosis (CF), 2001-2007. *Pediatric Pulmonology,* 46, 8, 770-776.

Carroll, A. E. & Downs, S. M. 2006. Comprehensive cost-utility analysis of newborn screening strategies. *Pediatrics,* 117, 5 Pt 2, S287-295.

Center for Jewish Genetics. 2014. *Usher Syndrome, Type 1F and Type III.* [Online]. Chicago, IL: Center for Jewish Genetics. Available: https://www.jewishgenetics.org/usher-syndrome-type-1f-and-type-iii [Accessed 1 August 2014].

Chou, J. Y., Matern, D., Mansfield, B. C. & Chen, Y. T. 2002. Type I glycogen storage diseases: disorders of the glucose-6-phosphatase complex. *Current Molecular Medicine,* 2, 2, 121-143.

Ciaccio, M. F., Wagner, J. P., Chuu, C. P., Lauffenburger, D. A. & Jones, R. B. 2010. Systems analysis of EGF receptor signaling dynamics with microwestern arrays. *Nature Methods,* 7, 2, 148-155.

Counsyl 2010. *The Universal Genetic Test - Disease Book v1.0.55,* Redwood City, CA, Counsyl, Inc.

Counsyl. 2014. *Lipoamide Dehydrogenase Deficiency.* [Online]. South San Francisco, CA: Counsyl, Inc. Available: https://www.counsyl.com/services/family-prep-screen/diseases/lipoamide-dehydrogenase-deficiency/ [Accessed July 28 2014].

D'haeseleer, P., Liang, S. & Somogyi, R. 2000. Genetic network inference: from co-expression clustering to reverse engineering. *Bioinformatics,* 16, 8, 707-726.

Edelmann, L., Dong, J., Desnick, R. J. & Kornreich, R. 2002. Carrier screening for mucolipidosis type IV in the American Ashkenazi Jewish population. *American Journal of Human Genetics,* 70, 4, 1023-1027.

Ekstein, J., Rubin, B. Y., Anderson, S. L., Weinstein, D. A., Bach, G., Abeliovich, D., Webb, M. & Risch, N. 2004. Mutation frequencies for glycogen storage disease Ia in the Ashkenazi Jewish population. *American Journal of Medical Genetics. Part A,* 129A, 2, 162-164.

Frick, K. D., Gower, E. W., Kempen, J. H. & Wolff, J. L. 2007. Economic impact of visual impairment and blindness in the United States. *Archives of Ophthalmology,* 125, 4, 544-550.

Genetics Home Reference. 2014. *Niemann-Pick disease.* [Online]. Bethesda, MD: Genetics Home Reference, U.S. National Library of Medicine. Available: http://ghr.nlm.nih.gov/condition/niemann-pick-disease [Accessed 28 July 2014].

Hallam, S., Nelson, H., Greger, V., Perreault-Micale, C., Davie, J., Faulkner, N., Neitzel, D., Casey, K., Umbarger, M. A., Chennagiri, N., Kramer, A. C., Porreca, G. J. & Kennedy, C. J. 2014. Validation for clinical use of, and initial clinical experience with, a novel approach to population-based carrier screening using high-throughput, next-generation DNA sequencing. *Journal of Molecular Diagnostics,* 16, 2, 180-189.

Heim, R. A., Sugarman, E. A. & Allitto, B. A. 2001. Improved detection of cystic fibrosis mutations in the heterogeneous U.S. population using an expanded, pan-ethnic mutation panel. *Genetics in Medicine,* 3, 3, 168-176.

Janecke, A. R., Mayatepek, E. & Utermann, G. 2001. Molecular genetics of type 1 glycogen storage disease. *Molecular Genetics and Metabolism,* 73, 2, 117-125.

Kaback, M. M. & Desnick, R. J. 1999 Mar 11 [Updated 2011 Aug 11]. *Hexosaminidase A Deficiency* In: Pagon, R. A., Adam, M. P., Ardinger, H. H. & Et Al., eds. GeneReviews® [Online]. Seattle, WA: University of Washington, 1993-2016. Available: http://www.ncbi.nlm.nih.gov/books/NBK1218/ [Accessed 28 July 2014].

Kaul, R., Gao, G. P., Aloya, M., Balamurugan, K., Petrosky, A., Michals, K. & Matalon, R. 1994. Canavan disease: mutations among Jewish and non-Jewish patients. *American Journal of Human Genetics,* 55, 1, 34-41.

Keats, B. J. B. & Lentz, J. 1999 Dec 10 [Updated 2013 Jun 20]. *Usher Syndrome Type I.* In: Pagon, R. A., Adam, M. P., Ardinger, H. H. & Et Al., eds. GeneReviews® [Online]. Seattle, WA: University of Washington; 1993-2016. Available: http://www.ncbi.nlm.nih.gov/books/NBK1265/ [Accessed 28 July 2014].

Khan, A., Ullah, S. & Nitz, J. 2011. Statistical modelling of falls count data with excess zeros. *Injury Prevention,* 17, 4, 266-270.

Ki, C. S., Han, S. H., Kim, H. J., Lee, S. G., Kim, E. J., Kim, J. W., Choe, Y. H., Seo, J. K., Chang, Y. J. & Park, J. Y. 2004. Mutation spectrum of the glucose-6-phosphatase gene and its implication in molecular diagnosis of Korean patients with glycogen storage disease type Ia. *Clinical Genetics,* 65, 6, 487-489.

Kutler, D. I., Singh, B., Satagopan, J., Batish, S. D., Berwick, M., Giampietro, P. F., Hanenberg, H. & Auerbach, A. D. 2003. A 20-year perspective on the International Fanconi Anemia Registry (IFAR). *Blood,* 101, 4, 1249-1256.

Lehavi, O., Aizenstein, O., Bercovich, D., Pavzner, D., Shomrat, R., Orr-Urtreger, A. & Yaron, Y. 2003. Screening for familial dysautonomia in Israel: evidence for higher carrier rate among Polish Ashkenazi Jews. *Genetic Testing,* 7, 2, 139-142.

Lines, C. 2013 (March). *Appraisal of screening the UK Ashkenazi Jewish population for Risk of Familial Dysautonomia.* [Online]. Oxford: Solutions for Public Health, National Health Service. Available: legacy.screening.nhs.uk/policydb_download.php?doc=213 [Accessed 28 July 2014].

Matalon, R. & Michals-Matalon, K. 1999 Sep 16 [Updated 2011 Aug 11]. *Canavan Disease.* In: Pagon, R. A., Adam, M. P., Ardinger, H. H. & Et Al., eds. GeneReviews® [Online]. Seattle, WA: University of Washington, 1993-2016. Available: http://www.ncbi.nlm.nih.gov/books/NBK1234/ [Accessed 28 July 2014].

National Institutes of Health (Nih). 1997 (April 14-16). *Genetic Testing for Cystic Fibrosis. NIH Consensus Statement No. 106; 15(4): 1-37.* [Online]. Bethesda, MD: National Institutes of Health. Available: http://consensus.nih.gov/1997/1997GeneticTestCysticFibrosis106html.htm [Accessed July 28 2014].

Nelson, W. B., Swint, J. M. & Caskey, C. T. 1978. An economic evaluation of a genetic screening program for Tay-Sachs disease. *American Journal of Human Genetics,* 30, 2, 160-166.

Ness, S. L., Ben-Yosef, T., Bar-Lev, A., Madeo, A. C., Brewer, C. C., Avraham, K. B., Kornreich, R., Desnick, R. J., Willner, J. P., Friedman, T. B. & Griffith, A. J. 2003. Genetic homogeneity and phenotypic variability among Ashkenazi Jews with Usher syndrome type III. *Journal of Medical Genetics,* 40, 10, 767-772.

Nestorowicz, A., Wilson, B. A., Schoor, K. P., Inoue, H., Glaser, B., Landau, H., Stanley, C. A., Thornton, P. S., Clement, J. P. T., Bryan, J., Aguilar-Bryan, L. & Permutt, M. A. 1996. Mutations in the sulonylurea receptor gene are associated with familial hyperinsulinism in Ashkenazi Jews. *Human Molecular Genetics,* 5, 11, 1813-1822.

Ouyang, L., Grosse, S. D., Amendah, D. D. & Schechter, M. S. 2009. Healthcare expenditures for privately insured people with cystic fibrosis. *Pediatric Pulmonology,* 44, 10, 989-996.

Palomaki, G. E., Haddow, J. E., Bradley, L. A. & Fitzsimmons, S. C. 2002. Updated assessment of cystic fibrosis mutation frequencies in non-Hispanic Caucasians. *Genetics in Medicine,* 4, 2, 90-94.

Park, N. J., Morgan, C., Sharma, R., Li, Y., Lobo, R. M., Redman, J. B., Salazar, D., Sun, W., Neidich, J. A. & Strom, C. M. 2010. Improving accuracy of Tay Sachs carrier screening of the non-Jewish population: analysis of 34 carriers and six late-onset patients with HEXA enzyme and DNA sequence analysis. *Pediatric Research,* 67, 2, 217-220.

Radhakrishnan, M., Van Gool, K., Hall, J., Delatycki, M. & Massie, J. 2008. Economic evaluation of cystic fibrosis screening: a review of the literature. *Health Policy,* 85, 2, 133-147.

Rake, J. P., Visser, G., Labrune, P., Leonard, J. V., Ullrich, K. & Smit, G. P. 2002. Glycogen storage disease type I: diagnosis, management, clinical course and outcome. Results of the European Study on Glycogen Storage Disease Type I (ESGSD I). *European Journal of Pediatrics,* 161 Suppl 1, S20-34.

Ramirez, A. 2006 Aug 24. Insurance Company Yields in Fight With Parents of a Tay-Sachs Child. Available at http://www.nytimes.com/2006/08/24/nyregion/24disease.html?_r=0. *The New York Times*

Schuchman, E. H. 2007. The pathogenesis and treatment of acid sphingomyelinase-deficient Niemann-Pick disease. *Journal of Inherited Metabolic Disease,* 30, 5, 654-663.

Schuchman, E. H. & Miranda, S. R. 1997. Niemann-Pick disease: mutation update, genotype/phenotype correlations, and prospects for genetic testing. *Genetic Testing,* 1, 1, 13-19.

Scott, S. A., Edelmann, L., Liu, L., Luo, M., Desnick, R. J. & Kornreich, R. 2010. Experience with carrier screening and prenatal diagnosis for 16 Ashkenazi Jewish genetic diseases. *Human Mutation,* 31, 11, 1240-1250.

Shaag, A., Saada, A., Berger, I., Mandel, H., Joseph, A., Feigenbaum, A. & Elpeleg, O. N. 1999. Molecular basis of lipoamide dehydrogenase deficiency in Ashkenazi Jews. *American Journal of Medical Genetics,* 82, 2, 177-182.

Simonaro, C. M., Desnick, R. J., Mcgovern, M. M., Wasserstein, M. P. & Schuchman, E. H. 2002. The demographics and distribution of type B Niemann-Pick disease: novel mutations lead to new genotype/phenotype correlations. *American Journal of Human Genetics,* 71, 6, 1413-1419.

Strauss, K. A., Puffenberger, E. G. & Morton, D. H. 2006 Jan 30 [Updated 2013 May 9]. *Maple Syrup Urine Disease.* In: Pagon, R. A., Adam, M. P., Ardinger, H. H. & Et Al., eds. GeneReviews® [Online]. Seatlle WA: University of Washington, 1993-2016. Available: http://www.ncbi.nlm.nih.gov/books/NBK1319/ [Accessed 28 July 2014].

Strom, C. M., Crossley, B., Redman, J. B., Quan, F., Buller, A., Mcginniss, M. J. & Sun, W. 2004. Molecular screening for diseases frequent in Ashkenazi Jews: lessons learned from more than 100,000 tests performed in a commercial laboratory. *Genetics in Medicine,* 6, 3, 145-152.

Terfve, C. & Saez-Rodriguez, J. 2012. Modeling signaling networks using high-throughput phospho-proteomics. *Advances in Experimental Medicine and Biology,* 736, 19-57.

Wang, Y. C., Lin, C., Chuang, M. T., Hsieh, W. P., Lan, C. Y., Chuang, Y. J. & Chen, B. S. 2013. Interspecies protein-protein interaction network construction for characterization of host-pathogen interactions: a Candida albicans-zebrafish interaction study. *BMC Systems Biology,* 7, 79.

Warren, E., Anderson, R., Proos, A. L., Burnett, L. B., Barlow-Stewart, K. & Hall, J. 2005. Cost-effectiveness of a school-based Tay-Sachs and cystic fibrosis genetic carrier screening program. *Genetics in Medicine,* 7, 7, 484-494.

Wong, L. J., Wang, J., Zhang, Y. H., Hsu, E., Heim, R. A., Bowman, C. M. & Woo, M. S. 2001. Improved detection of CFTR mutations in Southern California Hispanic CF patients. *Human Mutation,* 18, 4, 296-307.
