# Supplementary figures and images for: Carrier screening by next‐generation sequencing: health benefits and cost effectiveness
Source: Mol Genet Genomic Med. 2016 Jan 29;4(3):292–302. doi: 10.1002/mgg3.204 (PMC4867563; doi:10.1002/mgg3.204)

## Total Cases Averted

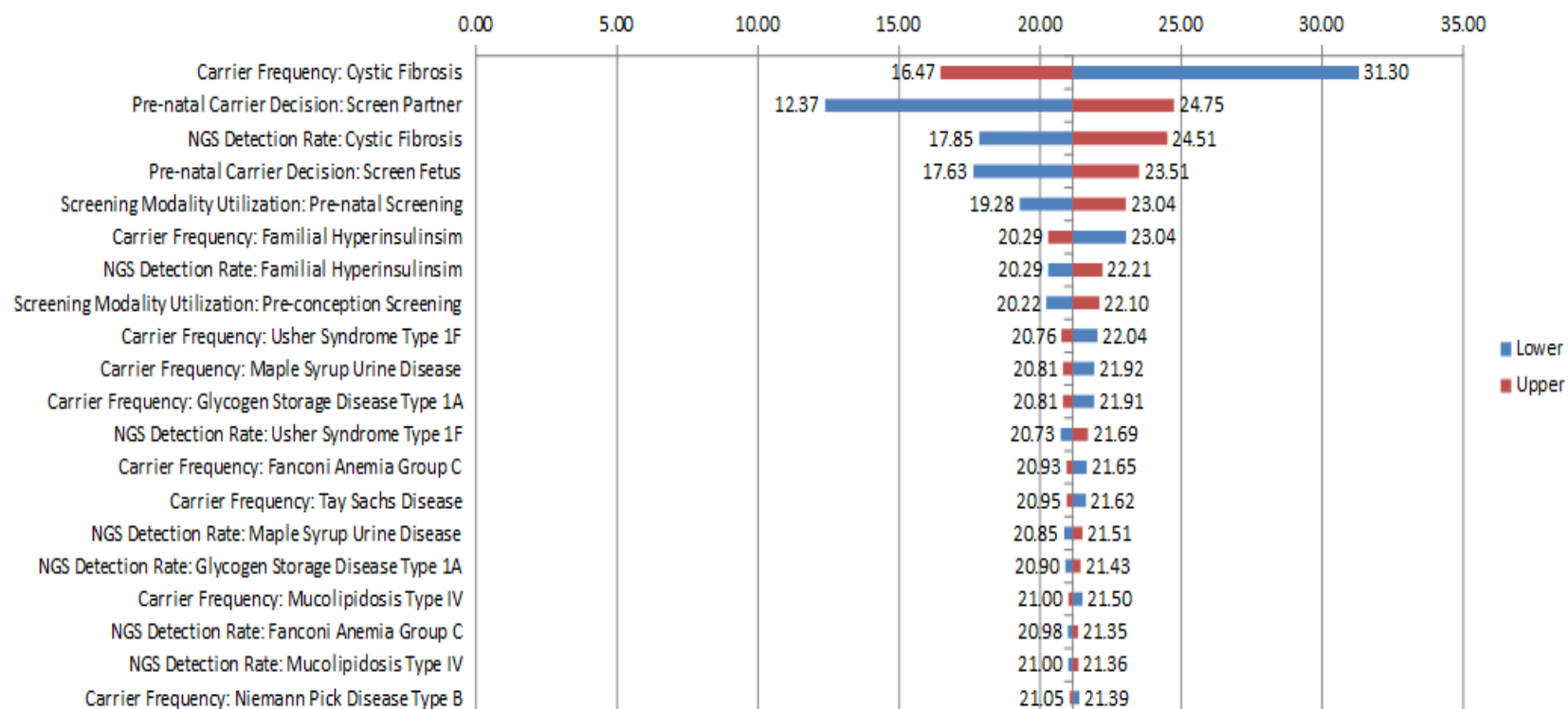

Supplement: Supplementary file 2 [file MGG3-4-292-s002.pdf]

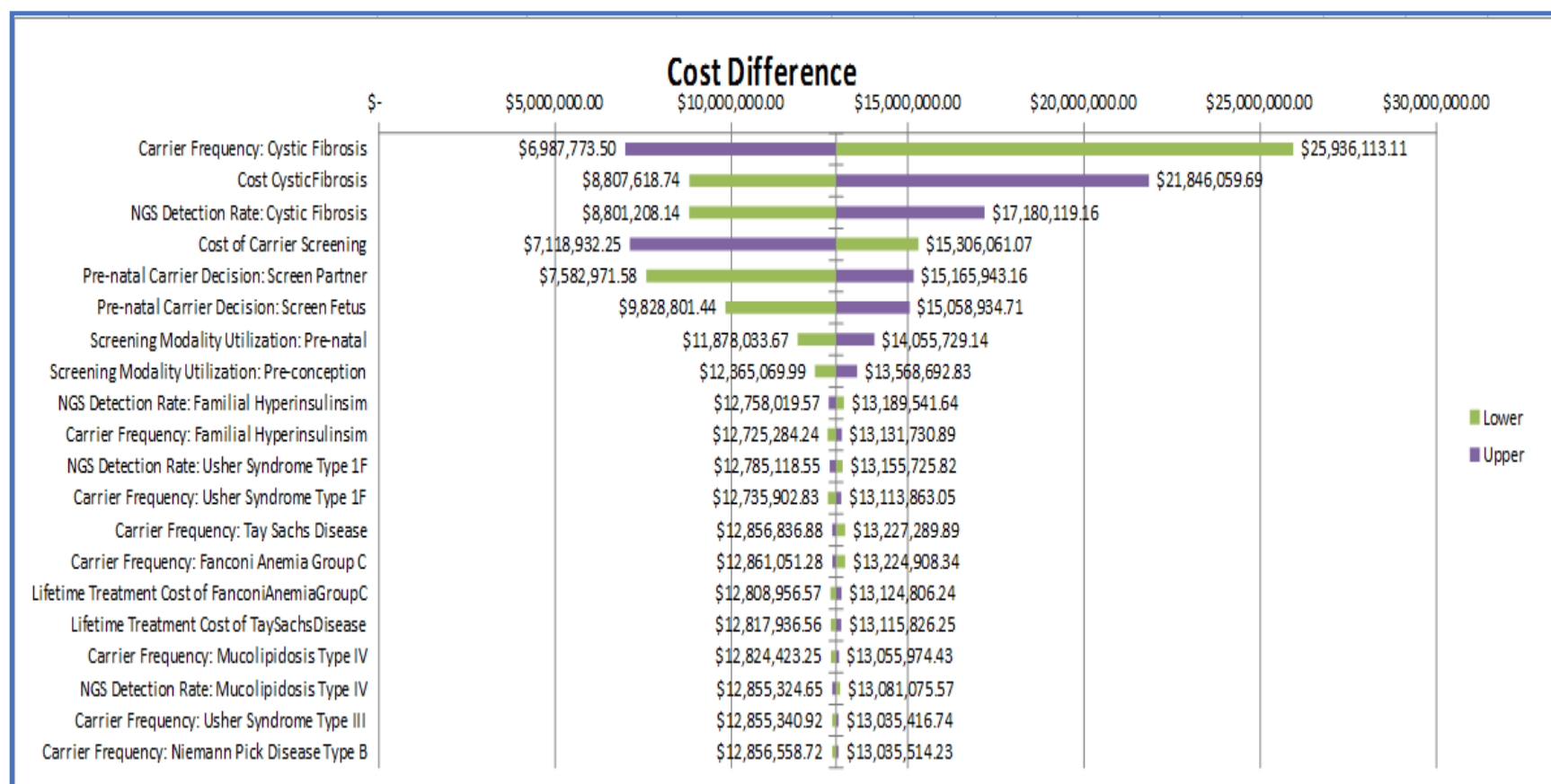

Supplement: Supplementary file 3 [file MGG3-4-292-s003.pdf]

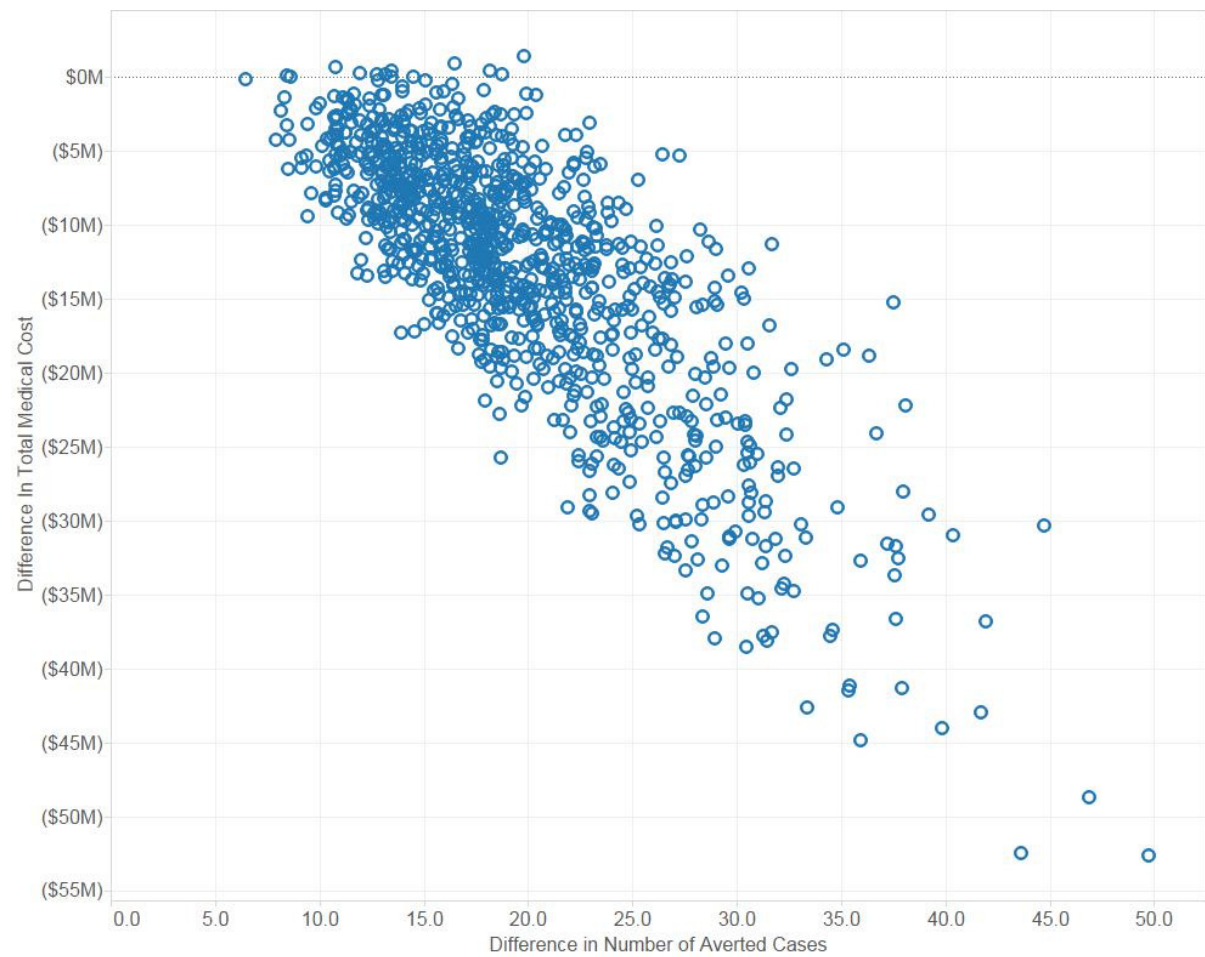

Supplement: Supplementary file 4 [file MGG3-4-292-s004.pdf]
